# Dynamic Metabolic and Transcriptional Responses of Proteasome-Inhibited Neurons

**DOI:** 10.3390/antiox12010164

**Published:** 2023-01-10

**Authors:** Ilinca Suciu, Johannes Delp, Simon Gutbier, Anna-Katharina Ückert, Anna-Sophie Spreng, Philipp Eberhard, Christiaan Karreman, Falk Schreiber, Katrin Madjar, Jörg Rahnenführer, Ivana Celardo, Ivano Amelio, Marcel Leist

**Affiliations:** 1 In Vitro Toxicology and Biomedicine, Department Inaugurated by the Doerenkamp-Zbinden Foundation, University of Konstanz, 78457 Konstanz, Germany; 2 Graduate School of Chemical Biology, University of Konstanz, 78457 Konstanz, Germany; 3 Graduate School of Biological Sciences, University of Konstanz, 78457 Konstanz, Germany; 4 Department of Computer and Information Science, University of Konstanz, 78457 Konstanz, Germany; 5 Faculty of Information Technology, Monash University, Clayton 3800, Australia; 6 Department of Statistics, TU Dortmund University, 44221 Dortmund, Germany; 7 Division for Systems Toxicology, Department of Biology, University of Konstanz, 78457 Konstanz, Germany; 8 The Center for Alternatives to Animal Testing in Europe, University of Konstanz, 78457 Konstanz, Germany

**Keywords:** proteasome inhibition, neurotoxicity, TempO-Seq, transcriptomics, metabolomics, proteostasis, Nrf2, oxidative stress, ATF4, LUHMES

## Abstract

**Highlights:**

**What are the main findings?**

- Detailed early neuronal response to the proteasome inhibitor MG-132
- Determination of sequence of events using multi-time-point multi-omics
- Recording of neuronal counter-regulations to stress over time
- Observation of rapid metabolic effects following proteasome inhibition

**Abstract:**

Proteasome inhibition is associated with parkinsonian pathology in vivo and degeneration of dopaminergic neurons in vitro. We explored here the metabolome (386 metabolites) and transcriptome (3257 transcripts) regulations of human LUHMES neurons, following exposure to MG-132 [100 nM]. This proteasome inhibitor killed cells within 24 h but did not reduce viability for 12 h. Overall, 206 metabolites were changed in live neurons. The early (3 h) metabolome changes suggested a compromised energy metabolism. For instance, AMP, NADH and lactate were up-regulated, while glycolytic and citric acid cycle intermediates were down-regulated. At later time points, glutathione-related metabolites were up-regulated, most likely by an early oxidative stress response and activation of NRF2/ATF4 target genes. The transcriptome pattern confirmed proteostatic stress (fast up-regulation of proteasome subunits) and also suggested the progressive activation of additional stress response pathways. The early ones (e.g., HIF-1, NF-kB, HSF-1) can be considered a cytoprotective cellular counter-regulation, which maintained cell viability. For instance, a very strong up-regulation of AIFM2 (=FSP1) may have prevented fast ferroptotic death. For most of the initial period, a definite life–death decision was not taken, as neurons could be rescued for at least 10 h after the start of proteasome inhibition. Late responses involved p53 activation and catabolic processes such as a loss of pyrimidine synthesis intermediates. We interpret this as a phase of co-occurrence of protective and maladaptive cellular changes. Altogether, this combined metabolomics–transcriptomics analysis informs on responses triggered in neurons by proteasome dysfunction that may be targeted by novel therapeutic intervention in Parkinson’s disease.

## 1. Introduction

Disturbed proteostasis is one of the key events in the pathological sequence leading to dopaminergic cell death and parkinsonian motor deficits [1,2]. Nevertheless, it is still not clear how changes in proteasome activity translate to the regulation of neuronal metabolism and mRNA pools. The elucidation of the hierarchy of events is difficult for complex diseases such as Parkinson’s disease (PD), where disturbed proteostasis, mitochondrial dysfunction and oxidative stress are interconnected [3,4,5,6,7]. However, there is solid genetic evidence for a causal role of perturbed protein degradation [8,9]. Several missense mutations and deletions in genes involved in protein turnover have been identified in patients with familial forms of PD. They include two genes involved in tagging proteins with the degradation signal ubiquitin (PARK2/Parkin, PARK15/FBXO7), the mitophagy-related kinase PARK6/Pink1 [7,10], the peptidase PARK5/UCHL1 and the autophagy regulator PARK7/VPS35 [11,12]. Furthermore, there is evidence from idiopathic PD on disturbed structure and function of the proteasome [8,11].

Besides proteasomal degradation, neurons use multiple pathways for protein degradation. These include lysosomal proteolysis and several autophagic pathways [13]. The multiple proteolytic routes are not redundant, as there are many proteins only degraded by the proteasome. The high specificity of this pathway is due to a specific labelling of targeted proteins by ubiquitin [14,15].

Compared to several other cell types, neurons have a high protein turnover. This is reflected by typical neurodegenerative phenotypes of autophagy-deficient mice [16,17] and by a particular sensitivity to proteasome inhibitors. For instance, a typical side effect of chemotherapy with proteasome-blocking drugs are severe neuropathies [18,19]. Moreover, proteasome inhibitors have been used to deplete dopaminergic neurons of rodents in ways similar to those observed in human PD [8,20,21].

Neurodegeneration can also be triggered by low concentrations of proteasome inhibitors in cell cultures of rodent and human neurons [22,23,24]. A particularly well-characterized in vitro model used is human LUHMES neurons. They can be cultured as fully post-mitotic neural networks, and they express the typical dopaminergic markers tyrosine hydroxylase (TH), as well as the plasma membrane and vesicular dopamine transporters DAT and VMAT2 [25,26,27,28]. LUHMES neurons treated with the reversible proteasome inhibitor MG-132 (carbobenzoxyl-l-leucyl-l-leucyl-leucine, MG) underwent apoptosis, preceded by a proteostatic stress response characterized by induction of the activating transcription factor 4 (ATF4). Moreover, up-regulation of the nuclear factor erythroid 2-related factor 2 (NRF2) indicated an oxidative stress response [24].

The loss of dopaminergic neurons in PD patients is a very slow process and it is presumed to take many years until the cells die. Early effects that precede cell death are of great interest, as this knowledge may eventually be used to slow or halt neurodegeneration. The complexity and heterogeneity of the human brain has made mechanistic studies on patients or post-mortem material very challenging. For this reason, several experimental approaches have been used, including yeast, Drosophila melanogaster, mammalian cells and mouse models [29,30]. Some studies suggested that the lethal effects of proteasome inhibition can be rescued by amino acid supplementation [29]. In this context, cysteine (Cys), a key amino acid involved in glutathione (GSH) synthesis, has been shown to be of particular importance [24]. Although there is accumulating evidence that proteasome inhibition affects not only protein levels, but also cell metabolism, studies addressing global metabolic effects due to reduced proteasome activity are still scarce.

The present study was designed to fill this gap of knowledge. It aimed to better understand of how proteasome impairment translates into metabolic changes in dopaminergic neurons. To this end, LUHMES neurons were treated with MG-132. The inhibitor concentration (100 nM) was chosen to have no effect on viability during the time window of sampling (up to 12 h), but to kill the cells after an additional 12 h [24]. Metabolome and transcriptome samples were obtained to track the neuronal adaptation to an impaired proteasome function. We focused on a comparison of early and late changes to identify a sequence of events. The overall changes pointed to initial metabolic and proteostatic stress that then triggered canonical stress responses and cell adaptations. Enhancement of such protective responses may be a novel strategy to reduce brain damage during neurodegenerative diseases.

## 2. Materials and Methods

### 2.1. Materials

Unless specified otherwise, cell culture consumables and media were from Gibco/Thermo Fisher Scientific (Waltham, MA, USA).

### 2.2. LUHMES Cell Culture

The LUHMES cells make up a homogenous dopaminergic population, with dopamine levels measured [26], pronounced DAT activity [26,31], cAMP (medium constituent)-dependent up-regulation of TH on both RNA and protein level [26]. Cells are fully sequenced and genetically intact, with all major dopaminergic markers [27].

As described previously [26], proliferating LUHMES cells were maintained in PM medium (i.e., Adv. DMEM/F12 supplemented with 2 mM L-glutamine, 1× N2 supplement (Invitrogen, Waltham, MA, USA) and 40 ng/mL recombinant human basic fibroblast growth factor (Bio-Techne, Minneapolis, MN, USA). Cells were kept in a humidified 5% CO_2_/95% air incubator at 37 °C and passaged every second day by trypsinization using 0.05% trypsin/EDTA (Invitrogen). To start differentiation, the medium was changed to DM (Adv. DMEM/F12 supplemented with 2 mM L-glutamine (Gibco, Rockville, MD, USA), 1 × N2 supplement (Invitrogen/Thermo Fisher Scientific, Waltham, MA, USA), 1 mM N6,2′-O-dibutyryl 3′,5′-cyclic adenosine monophosphate (cAMP), 1 µg/mL tetracycline (Sigma-Aldrich, Merck, Darmstadt, Germany) and 2 ng/mL recombinant human glial cell-derived neurotrophic factor (GDNF, Bio-Techne, Minneapolis, MN, USA). Cell culture plates and flasks (Sarstedt, Nümbrecht, Germany) were pre-coated with 1 μg/mL fibronectin and 50 μg/mL poly-L-ornithine (PLO) (Sigma-Aldrich).

### 2.3. LUHMES Differentiation and Exposure Scheme

The differentiation of LUHMES cells was performed as previously described [32,33,34]. For the transcriptomics experiment and ATP analysis, cells differentiated for 48 h (day 2, d2) were re-seeded into 96-well plates at a density of 200,000 cells/cm^2^ in DM and matured until day 6. Subsequently, a reverse treatment with 100 nM MG-132 (Selleckchem, Houston, TX, USA) was applied to the cells, with exposure times ranging between 0 and 12 h. All the samples were collected simultaneously at the end of the experiment.

For the metabolomics experiment, for amino acid profiling and for Western blotting, the d2 re-seeding was conducted in 10 cm dishes at a density of 200,000 cells/cm^2^. Cells in these plates were further differentiated until day 6, when they were treated with 100 nM MG-132 for different times. The control samples, also referred to as 0 h MG, were treated with 0.1% dimethyl sulfoxide (DMSO, Merck, Darmstadt, Germany) for 12 h. In parallel experiments, 1 mM L-cysteine (Sigma-Aldrich) was added to cells at different times after MG-132, to check if the cells could still be rescued.

### 2.4. Sample Preparation for the Metabolomics Experiment

Cell culture dishes were processed for metabolomics analysis at the indicated time points for each test condition (3, 6, 9, 10.5 and 12 h). The controls were treated with DMSO at 0 h and collected at 12 h. First, the supernatant was removed and a wash with 1 x PBS was performed, followed by re-suspension in 80% methanol using cell scrapers. The residual cell lysate was subsequently collected in a second scraping step in 80% methanol and added to the previously harvested samples. Lysates were then flash-frozen in liquid N_2_ and stored at −80 °C until the next step. Prior to centrifugation (30 min, 20,000× *g*, 4 °C), the samples were thawed and homogenized by shaking. After separation, the supernatant was transferred in a new Eppendorf tube, then placed in a Savant SpeedVac concentrator (Thermo Fisher Scientific, Waltham, MA, USA) for drying, whereas the methanol-precipitated pellet was used for the measurement of protein content through the Bradford assay. The dry samples were further stored at −80 °C until shipment.

### 2.5. Metabolomics Profiling

Samples were stored at −80 °C until they were processed by Metabolon (Research Triangle Park, NC, USA). A non-targeted approach was employed to generate the metabolic profiles utilizing UPLC (ultra-performance liquid chromatography) coupled with MS/MS (tandem mass spectrometry). In order to detect a multitude of metabolites with wide-ranging physico-chemical properties, 4 different methods were used in parallel: (1) reverse phase (RP)/UPLC-MS/MS under positive ionization, (2) RP/UPLC-MS/MS under positive ionization optimized for more hydrophobic compounds, (3) RP/UPLC-MS/MS under negative ionization and (4) HILIC/UPLC-MS/MS (negative ionization). All analyses were conducted using a Waters ACQUITY ultra-performance liquid chromatography (UPLC) and a Thermo Scientific Q-Exactive high resolution/accurate mass spectrometer interfaced with a heated electrospray ionization (HESI-II) source and Orbitrap mass analyzer operated at 35,000 mass resolution. The instrument used dynamic exclusion to alternate between MS and MS/MS scans and covered a range of 70–1000 *m/z*.

Data extraction, peak identification and quality controls were performed by Metabolon using its own hardware and software. The identification of metabolites was achieved by the Laboratory Information Management System (LIMS), which compared the paired retention time/index (RI)–mass-to-charge ratio (*m/z*) against a library of more than 3300 purified standards.

Data analysis and visualization was conducted using the R software. For each sample, the metabolite data were normalized to the average of the DMSO controls. The statistical analysis was conducted using a moderated *t*-test statistics implemented by the R package limma [35]. The Benjamini–Hochberg method was applied to correct for multiple testing. Metabolites with an adjusted *p* value < 0.05 were considered significant. The complete data matrix can be found in Supplementary Table S1.

### 2.6. LDH Release Assay

LDH activity was determined from the medium of the same samples used for the metabolomics experiment, using a method described earlier [24]. Briefly, a volume of 180 µL reaction buffer containing NADH (100 μM) and sodium pyruvate (600 μM) in potassium phosphate buffer (pH 7.4) was mixed with 20 µL of sample medium. In the following 15 min, the absorption change at 340 nm was measured. The average slope of NADH consumption was calculated and used to quantify the LDH release in MG treatments relative to the DMSO control.

### 2.7. ATP Measurement

Intracellular ATP was determined as described earlier [32,36]. Briefly, cells were lysed by adding to the medium the luciferase-containing CellTiterGlo 2.0 mix (Promega, Madison, WI, USA). The data obtained from measuring luminescence were background-corrected, then normalized to DMSO controls.

### 2.8. Resazurin Reduction Assay

Resazurin (Sigma-Aldrich) was added to the cell culture medium to a final concentration of 2 μg/mL. After incubation for 60 min at 37 °C, the fluorescence signal was measured (530 nm_ex_, using a 590 nm_em_). After background subtraction, fluorescence values were normalized to the average fluorescence of DMSO controls.

### 2.9. Image Analysis of Viable Cells

Image acquisition of LUHMES cells live-stained with 1 µg/mL Hoechst H-33342 and 1 µM Calcein-AM was conducted by an automated high-content imager (Cellomics, Waltham, MA, USA), as described previously [25,33,34].

### 2.10. Mitochondrial Respiration Function

LUHMES cells were plated on d2 in PLO/fibronectin-coated “Seahorse 24-well plates” (Agilent, Santa Clara, CA, USA) at a density of 1,000,000 cells/well. On d6, medium was replaced with Agilent Seahorse XF DMEM Medium, pH 7.4, supplemented with 18 mM glucose, 2 mM glutamine and 1 mM pyruvate. One hour later, basal mitochondrial oxygen consumption was first assessed using the Seahorse XFe24 Analyzer from Agilent (Santa Clara, CA, USA), then MG-132 was injected at a final concentration of 1 µM. Oxygen consumption was monitored for another 6 h. Subsequently, the Mito Stress Test was performed, by taking several measurements after injecting each of (i) 1 μM oligomycin, (ii) 1.5 μM carbonyl cyanide-4-(trifluoromethoxy)phenylhydrazone (FCCP) and (iii) 0.5 μM rotenone with 0.5 μM antimycin A. For each well, the total number of cells was quantified and used to normalize the oxygen consumption rate. Individual mitochondrial parameters were determined after normalization according to the manufacturer’s instructions.

### 2.11. Protein Determination

The total protein concentration was determined using a BCA protein assay kit (Pierce/Thermo Fisher Scientific, Rockford, IL, USA). The pellets obtained by methanol precipitation were re-suspended in 1 mL 100 mM NaOH and further incubated on a shaker at 50 °C overnight. For the BCA assay, samples were first diluted 1:10.

### 2.12. Western Blot Analysis

Cell lysis was performed in 1× Laemmli buffer [37], subsequently boiled at 95 °C for 5 min for protein denaturation. Removal of long DNA strands was conducted using the NucleoSpin Filters (Macherey-Nagel, Düren, Germany) in a short centrifugation step (1 min, 10,000× *g*, RT). The samples were used for denaturing gel electrophoresis by migration on 6–15% SDS-PAGE according to the molecular weight of the protein under analysis. For the transfer of the migrated proteins onto nitrocellulose membranes (Amersham, Buckinghamshire, UK), the iBlot 2 Gel Transfer Device (Invitrogen) was used. For blocking, membranes were incubated for 1 h with a solution of 5% BSA (*w/v*) in TBS-Tween (0.1% (*v/v*)). Next, overnight incubation with primary antibodies was performed at 4 °C on a shaker, followed by 3 washing steps with TBS-Tween (0.1%) and thereafter incubation with the horseradish peroxidase-conjugated secondary antibodies for 1 h at RT. After washing again 3 times, the ECL Western Blot substrate (Pierce/Thermo Fisher Scientific, Rockford, IL, USA) was applied to the membranes. Rabbit anti-ATF4 clone D4B8, mouse anti-ubiquitin clone P4D1, rabbit anti-NRF1 clone D5B10, rabbit anti-NRF2 clone D1Z9C, rabbit anti-PARP polyclonal (9542) antibodies were from Cell Signaling Technology (Danvers, MA, USA). Peroxidase AffiniPure goat anti-mouse IgG was from Jackson Immunoresearch (Cambridge, UK), anti-rabbit HRP antibody was from GE Healthcare (Chicago, IL, USA). The experiments were performed only once to confirm earlier data [24] under the conditions used for metabolomic sampling (large dishes). For this reason, relative band intensities were not quantified, and the data are given in graphical form only. Full original gels will be provided upon request. The molecular weights were estimated based on the Precision Plus Protein Dual Color standard from Bio-Rad (Hercules, CA, USA) and are marked in the figures.

### 2.13. Amino Acid Analysis

The amino acid analysis was conducted as described previously [32].Washing of the 10 cm cell culture dishes was performed once with PBS, followed by quenching with 50% *v/v* methanol/H_2_O. The resulting solution was incubated at 4 °C in an Eppendorf Thermomix (Hamburg, Germany) at 1400 rpm for 30 min, then centrifuged for 15 min at 21,000× *g* at 4 °C. The supernatant was transferred to new tubes and dried in the SpeedVac concentrator. Samples were re-suspended in 2% (*w/v*) 5-sulfosalicylic acid (SSA), then centrifuged for 20 min at 1440 rpm, 4 °C. The supernatant was centrifuged for 10 min at 20,000× *g*, 4 °C. Utilizing the Sykam S433 amino acid analyzer (Sykam, Fürstenfeldbruck, Germany), the separation of amino acids was carried out by HPLC (lithium-based anion exchange column: 7 µm diameter, 10% cross-links, cat# 5125022) and post-column derivatization with ninhydrin. Elution was performed using buffers with increasing pH (pH 2.9→pH 12), ion strength (buffer concentration 0.12–0.45 M) and using a temperature gradient. The reaction products were quantified at 570 nm (most amino acids) and at 440 nm (for the intermediate products in the case of cysteine and proline). The area under the peak was quantified using the ChromStar 7 software (SCPA, Weyhe-Leehste, Germany) and by comparison to reference standards, the concentration was determined.

### 2.14. Sample Preparation for the Transcriptomics Experiment

For sample preparation, the medium in 96-well plates was replaced by Biospyder Lysis Buffer (33 µL/well; Biospyder Tech., Glasgow, UK). Following a 15 min incubation at room temperature, the plates were sealed and frozen at −80 °C for lysis completion.

Targeted transcriptome sequencing (including QC, alignment and read quantification) was conducted at Bioclavis (Biospyder Tech., Glasgow, UK) using the TempO-Seq technology in combination with the EU-ToxRisk v2.1 probe panel [38]. For each of the 3257 targeted genes, a 50 bp fragment was amplified, while also introducing sample-specific barcodes, which subsequently enabled sample pooling for the next-generation sequencing of the collection. A reference library containing the collection of all amplification products was used for assigning read counts to each targeted gene. A pre-filtering step for library size (<0.2 million) and average gene count (<1.5) was performed. The counts per gene were normalized to counts per million by dividing by the total number of mapped reads per sample and multiplying by 10^6^. The effect of normalization was checked by boxplots and distribution plots (not shown) and no outlier samples were identified. The differential gene expression (DGE) analysis of each treatment against the control group was conducted by the Wald test implemented in DESeq2/R [39], including a FDR correction using the Benjamini–Hochberg algorithm. The complete data matrix can be found in Supplementary Table S2. To check for functional enrichment, we applied the WMEAN algorithm from decoupleR [40] on the gene expression statistics (stat) mapped onto DoRothEA regulons [41] and PROGENy pathway signatures [42].

### 2.15. Curve Fitting and Statistics

All samples analyzed in this study were prepared separately and treated as statistically independent (biological replicates). For the metabolomics and amino acid analysis, 3 treated samples per time point and 4 control samples were used. For transcriptomics analysis, 6 samples were produced and measured for each condition. If not mentioned otherwise, data displayed are the means ± SEM. Data of treated samples are expressed relative to solvent (DMSO) controls. Error bars are used as quantitative measure of data variation; in addition, individual data points are displayed where this makes the data structure more transparent. The plots were created using GraphPad Prism 7.0 (GraphPad Software, La Jolla, CA, USA).

## 3. Results

### 3.1. Experimental Design to Study the Inhibition of the Proteasome in Mature Dopaminergic Neurons

**Figure 1:**
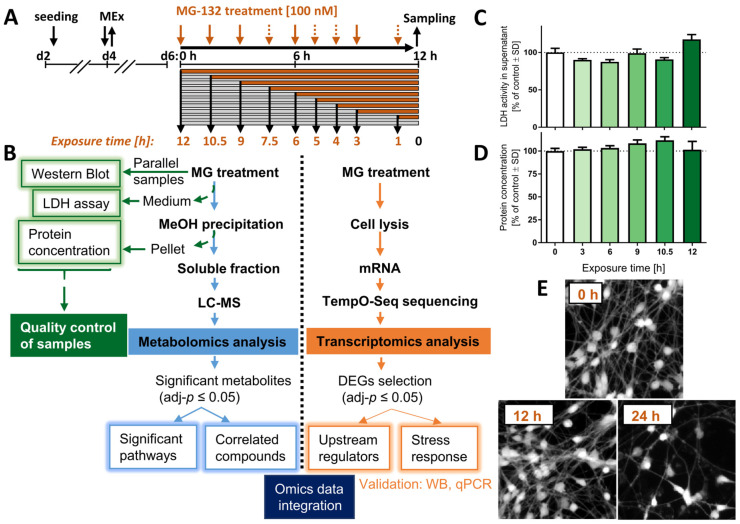
Experimental workflow and sample validation. LUHMES neurons were exposed to the proteasome inhibitor MG-132 (MG) for various times before samples were prepared. (**A**) Culture and treatment scheme: pre-differentiated LUHMES cells (d2) were plated, and medium was exchanged on d4 (MEx). At the stage of mature neurons (d6), cell cultures were treated with 100 nM MG for 1–12 h. Orange arrows indicate the time of treatment initiation in a reversed order. Dashed arrows: sampling time points only for the transcriptomics, but not metabolomics profiling. (**B**) Flow diagram of the experiment, including quality control assays (left), the metabolomics pipeline (middle) and of the transcriptomics procedure (right). (**C**) The cell viability was assessed by the lactate dehydrogenase (LDH) release assay. Cell supernatants were obtained from the same wells that were used for the metabolomics analysis. The activity of LDH in the medium was normalized to the one measured above untreated cells. (**D**) The protein content was measured from the pellet obtained by methanol (MeOH) precipitation during the preparation of the metabolomics samples. (**E**) LUHMES cells, treated with MG for 0, 12 and 24 h MG were stained with calcein-AM, and representative pictures are shown. Data shown in graphs are means ± SD of independent replicates. For MG treatments, 3 different samples were analyzed. For controls (DMSO), 4 replicates were prepared to provide for more robust baseline data. DEG, differentially expressed gene; LC-MS, liquid chromatography–mass spectrometry; MG, proteasome inhibitor MG-132.

To characterize the sequence of events triggered by proteasome inhibition in dopaminergic neurons, we conducted a dual omics time course study (Figure 1A). Three types of quality control were obtained, to confirm (i) previously identified cell signaling responses [24], (ii) unimpaired viability during the experimental process and (iii) equal protein content (=cell number) of samples. In the metabolomics workflow, differentially abundant small molecules were identified for each time point. Ultimately, the findings from transcriptomics and metabolomics data analyses were used together to pinpoint some of the major cell-regulatory modules affected.

The treatment was initiated on day 6 of differentiation (d6), when LUHMES cells display a dopaminergic phenotype, marked by expression of high levels of dopamine transporter (DAT/SLC6A3), tyrosine hydroxylase (TH) and Fox3/NeuN, as well as a complete cell cycle exit [26]. Cells were exposed to the proteasome inhibitor MG-132 (MG; 100 nM) for up to 12 h, and samples were obtained for metabolomics analysis, transcriptomics and quality controls at different time points (Figure 1B).

Up to 12 h after MG treatment, the levels of extracellular LDH did not increase compared to control conditions (Figure 1C). This is in line with our previous data showing that 100 nM MG induces death only at time points > 12 h, and then killing > 80% of LUHMES cells within 24 h [24]. By using the Bradford assay, we also confirmed that total protein levels for each test sample were similar (Figure 1D).

For the transcriptomics experiment, viability was verified by imaging cells stained with calcein-AM before the harvest (Figure 1E). Samples prepared at ≤12 h after MG treatment show a similar number of calcein-positive cells compared to control. In summary, control samples indicated that although most MG-treated cells died after 24 h exposure, all omics samples (1–12 h) were obtained from viable cell populations.

### 3.2. Internal Controls of Proteasome Inhibition

To verify that the neurons used for the omics sampling behaved as showed previously [24], markers for proteasome activity, proteotoxic and oxidative stress, as well as cell death, were assessed (Figure S1A). The rapid increase (3 h) of ubiquitinated proteins confirmed the successful inhibition of the proteasome. We also observed increased levels of the transcription factors Atf4, Nrf-1 and Nrf-2 within 3–6 h. The initiation of PARP proteolysis at approximately 10–12 h was well in line with earlier findings showing that the irreversible commitment of neurons to death (apoptosis) occurs in this time frame [24].

**Figure 2:**
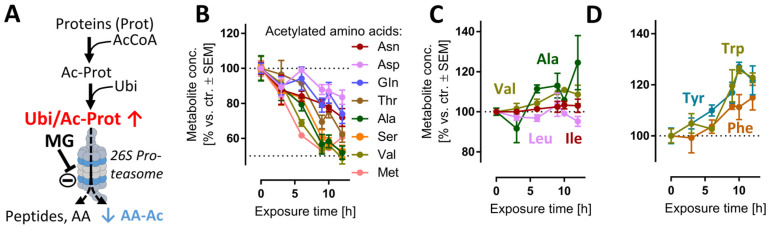
Reduced amounts of acetylated amino acids as evidence for proteasome inhibition. Cells were treated as described in Figure 1 to obtain time-dependent metabolomics data, following addition of 100 nM MG. Data on acetylated amino acids (AA-Ac) and some other amino acids (AAs) were extracted from the metabolome data matrix. (**A**) Chain of events leading to the generation of AA-Ac. Post-translational modification of proteins by the acetyl group leads to polypeptide chains containing AA-Ac. The ubiquitination (Ubi) of these proteins (Ubi/Ac-Prot) can lead to their degradation by the 26S proteasome. This process yields: amino acids, small peptides and AA-Ac. (**B**) Time course of the levels of AA-Ac over time. (**C**,**D**) Quantification of hydrophobic and aromatic AAs over time. Data are means ± SEM of independent replicates. For MG treatments, 3 different samples were analyzed. For controls (DMSO), 4 replicates were prepared to provide for more robust baseline data. AcCoA, acetyl coenzyme A; MG, proteasome inhibitor MG-132.

We reasoned that evidence for the impairment of the proteasome function may be obtained directly from metabolome samples. For instance, proteasomal degradation of N-acetylated proteins is the main metabolic pathway [43] to generate several N-acetylated amino acids (AA-Ac) (Figure 2A). Indeed, acetylated Asn, Asp, Gln, Thr, Ala, Ser, Val and Met were all depleted upon MG treatment (Figure 2B), while non-modified AAs remained stable (Figure 2C) or were even increased (Figure 2D). Another modified amino acid that is produced in mammalian cells only via post-translational protein modification and subsequent protein degradation is (6-N,6-N,6-N)-trimethyllysine (Kme3). This metabolite would be expected to be depleted upon cessation of proteasome function (Figure S1B), and this is exactly what we observed (Figure S1C).

Small peptides, which are also typical products of proteasome activity (Figure S1B), were particularly quickly decreased (after 3 h) (Figure S1D). One reason why depletion kinetics of AA-Ac and dipeptides differed may be linked to their respective downstream hydrolytic processes. Additionally, a decreased availability of the acetyl donor acetyl coenzyme A (acetyl-CoA) may contribute to this phenotype. Indeed, the levels of acetyl-CoA were reduced at >6 h, albeit not significantly (Figure S12B).

Another reason for the observed depletion of AA-Ac and dipeptides may be a generalized metabolite/AA loss. This was obviously not the case: for instance, the aromatic amino acids (Trp, Tyr, Phe) showed a sustained increase after 6 h. These AAs are involved in the transmission of nervous signals and quenching of reactive oxygen species (ROS) in the brain, among other processes [44]. Additionally, the levels of other hydrophobic amino acids (Val, Ala, Ile, Leu) did not decrease, but remained constant relative to the untreated control (Figure 2C).

Early metabolic changes were consistent with inhibition of proteasome activity. Hence, we decided to investigate the secondary metabolic alterations in the same experimental conditions.

### 3.3. Global Metabolome Changes in MG-Exposed Neurons

**Figure 3:**
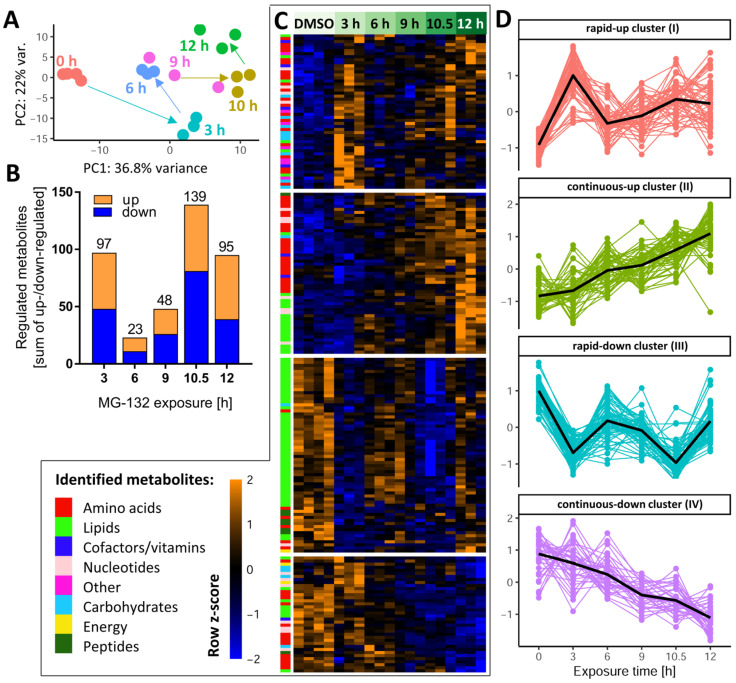
Dynamics of the MG-132-induced metabolic changes over time. Altogether, 386 cellular metabolites were quantifiable under the control condition. Based on LIMMA statistics, 206 of these metabolites were changed significantly at least at one time point by MG-132 treatment. A high-level overview of these metabolome changes is given here. (**A**) Principal component analysis (PCA) of all changed metabolites: the first two dimensions are plotted, with each analyzed sample displayed as filled circle (color-coded according to the sampling time). The axes were scaled according to the variance covered by the principal components (PCs). (**B**) The absolute numbers of metabolite changes for each exposure time are displayed on top of the bars. The colored segments indicate the numbers for the two directions of regulation: up (orange) and down (blue). (**C**) Unsupervised clustering of metabolite time-profiles. The heat map colors represent the z-scores of the row-wise normalized relative levels for each metabolite. All replicates and conditions are shown. A color code was used to visualize the major biochemical groupings (e.g., lipids, nucleotides) of the metabolites. The clustering dendrogram is not shown, but the four major clusters are indicated by white separating lines. (**D**) For each of the clusters, the time profiles of their associated metabolites are given. Clusters are named by the average time profile of their constituents (e.g., rapid-up or continuous-down). The metabolite z-score values were averaged per treatment condition in each cluster and are shown as black lines. Metabolites were considered to be regulated if the false discovery rate–corrected *p*-value for at least one time point was ≤0.05. DMSO, dimethyl sulfoxide.

To obtain an initial overview of the multidimensional metabolomics data set, we performed a principal component analysis (PCA) on the normalized data matrix of all 386 metabolites that were clearly identified and quantified by our approach. Overall, the PC plot showed a good agreement between the individual replicates and a clear separation of all time points (Figure 3A). Surprisingly, PCA revealed that neurons had a largely altered metabolic signature already after 3 h. It was also notable that, e.g., 10 h samples were clearly distinct from 12 h samples.

After this general overview, we determined for each time point the subset of metabolites which were different from the control samples (Supplementary Table S1). Already after 3 h, a quarter of all measurable metabolites were significantly de-regulated (Figure 3B), indicative of a fast cellular response. Approximately half of these metabolites were up-regulated. This confirmed that the proteasome inhibitor did not lead to a general metabolite down-regulation. In the time period of 6–9 h, the metabolic de-regulation was smaller than at 3 h, which may be due to cellular adaptations to stress. At later time points (10–12 h), large metabolic derangements were observed, possibly related to an incipient failure of cellular homeostasis. To uncover the time profiles of changes for each metabolite, we used hierarchical clustering of the metabolite level–time matrix. Four major clusters emerged (Figure 3C): one showed a fast up-regulation and another one a rapid down-regulation; the remaining two clusters showed a continuously increasing up- or down-regulation over time (Figure S2).

To obtain a measure for the “consistency” of regulation, we compared the early (3 h) and late (10.5 h) responses. Most compounds up-regulated at 3 h also had increased levels at 10.5 h, and metabolites depleted at 3 h were usually also lower than control levels at 10.5 h (Figure S3A,B). A scatter plot showed the general correlation of 3 h vs. 10.5 h changes (r^2^ = 0.18) (Figure S3C). Some notable deviations of this trend were observed for glutathione-related metabolites (over-proportional up-regulation with time), and for metabolites possibly related to impaired respiration (AMP and NADH, returning to control levels after prolonged exposure).

As a complementary approach to check the consistency of metabolic responses over time, we used Venn diagrams separately for up- and down-regulated compounds. Two metabolites, acetylcholine (ACh) and Kme3, were steadily decreased over the entire time course (Figures S4 and S5). The decrease in Kme3 might be a direct consequence of blocking the proteolytic degradation of proteins, since tri-methylation of Lys is a typical posttranslational modification of proteins, particularly histones [45]. A relatively high number of metabolites was consistently up-regulated at 9–12 h. They included some purines and amino acid derivatives. (Figure S5B,C).

Instead of looking at individual metabolites, we also considered the coordinate regulation of metabolite groups that were functionally connected (e.g., members of a metabolic pathway). Classical overrepresentation analysis (ORA) and metabolite set enrichment analysis (MSEA) gave significant results at both early and late time points. For example, at 3 h, the pentose phosphate pathway and glycolysis were significantly perturbed (Figure S6A), both important for energy metabolism. The majority of enriched metabolite sets disturbed at 6–12 h were related to lipid metabolism. At later time points (10–12 h), pyrimidine metabolism was significantly affected (Figure S6B).

### 3.4. Global Transcriptome Changes in MG-Exposed Neurons

**Figure 4:**
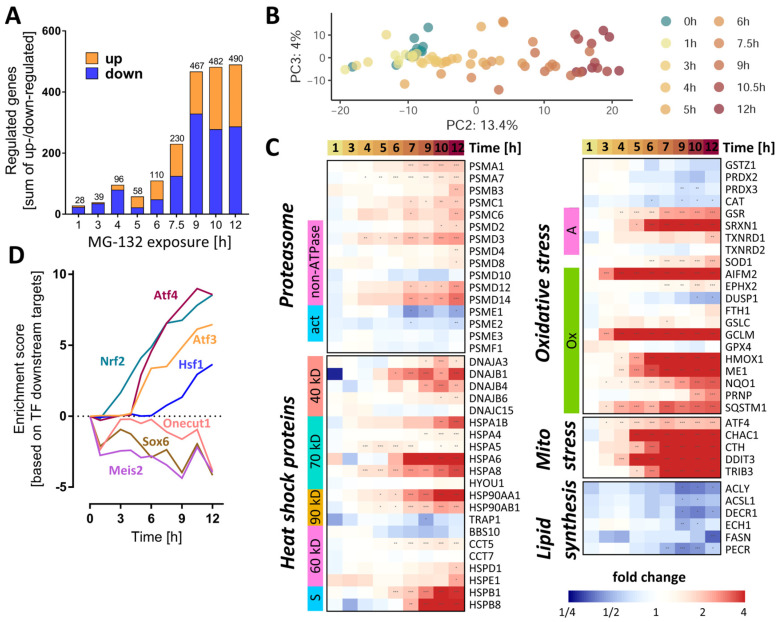
Dynamics of the MG-132-induced transcriptomic changes over time. Transcriptome data were obtained from LUHMES cells treated with MG-132 (100 nM) for 1–12 h, as outlined in Figure 1. Significant transcriptomic changes were identified by performing a differential gene expression analysis for each MG exposure time relative to the DMSO control. As output metrics, we provide the average log2 fold changes for each of the transcripts, including the standard deviation, and the statistical significance of the change. (**A**) Principal component analysis (PCA) second and third dimensions are displayed, scaled according to the variances covered. The first dimension captured mostly background noise and was, therefore, not considered. Each dot represents a time point (label) based on data from averaged replicates. (**B**) The absolute numbers of differentially expressed genes per exposure time are displayed. The two bar sections indicate the numbers for the two directions of regulation: up (orange) and down (blue). (**C**) The heat map shows the measured changes in transcript abundance (log2 fold changes) for members of the heat shock protein family (40/60/70/90 kD, types of heat shock proteins based on their molecular weights, s, small chaperones;), proteasome subunits (ATP, ATPase subunits; α/β, components of the α/β proteasome rings making up the 20S core proteasome; act, proteasome activators; i, proteasome inhibitors), oxidative stress responsive genes (A, other antioxidants; G, glutathione peroxidases; Op, other peroxidases; Ox, oxidative stress responsive genes; P, peroxiredoxins; S, superoxide dismutases) and lipid synthesis pathways. (**D**) Overrepresentation analysis of binding sites for transcription factors on MG-132-regulated genes: protein activity predictions were made for each individual time point for master regulators (DoRothEA database regulons). A subset of perturbed transcription factors is shown. The enrichment scores are row-wise normalized to display more clearly the time profile of their effect on the transcriptome. The results of the full collection of regulators analyzed are displayed in Supplementary Figure S10. Additionally, Table S2 contains further information about these DEGs (full gene names and enzyme identifiers). Transcriptomics samples were prepared separately and treated as statistically independent (biological replicates). For each condition, 6 samples were produced and measured. * adjusted-*p* < 0.05; ** adjusted-*p* < 0.01; *** adjusted-*p* < 0.001; MG, proteasome inhibitor MG-132.

The expression of 3257 unique genes (3565 probes) was quantified for eleven different MG exposure times, ranging from 0 to 12 h. The number of differentially expressed genes (DEGs) (Figure 4A) showed a time-dependent increase, reaching a saturation level around 500 genes at ≥9 h. To obtain an initial overview of the time-dependent transcriptome dynamics, a PCA was used (Figure 4B). PC2 captured a time-dependent clustering of the samples, with the samples corresponding to the longest exposure times located furthest away from the untreated controls.

As a first approach to detect coordinated regulation patterns, we examined functionally related groups of genes important for proteostasis. For instance, several proteasome subunits (PSM) already had an increased gene expression after 6 h (Figure 4C). Their upstream regulator, NRF1, also showed a higher (protein) expression after ≥3 h (Figure S1A).

We next checked for functional enrichment of transcription factors (TF). Based on this analysis, the activities of the master redox homeostasis regulator Nrf2 and of Atf4 were already boosted at ≥3 and 5 h, respectively, and showed a steady increase thereafter (Figure 4D and Figure S7). From 6 h onwards, the activities of Atf3 and of heat shock factor 1 (Hsf1) were also predicted to continuously rise. By contrast, Onecut1, Meis2 and its target Sox6 were estimated to decline already after 1 h MG, with a subsequent oscillating trend.

A closer look at the heat shock protein family of chaperones and chaperonins revealed a collectively elevated transcription after 6–7 h MG (Figure 4C). To assess the cell’s response strategy to oxidative stress, we investigated the levels of antioxidative genes and found some important players (NQO1, HMOX1, GCLM, AIFM2) to be up-regulated within a time window of 3–6 h after MG treatment (Figure 4C). Likewise, key effectors of the mitochondrial stress response regulated by Atf4 (CHAC1, CTH, DDIT3, TRIB3) had higher levels of transcripts starting with ~ 4 h after MG. Moreover, since the metabolite set analysis highlighted changes in lipid metabolism, we assessed the gene expression levels of enzymes involved in lipid synthesis. Those meeting the significance criteria (at any time point) showed a rather late (≥9 h) downwards trend, suggesting a decrease in lipogenesis.

Analysis of the activity of the transcription factor TP53 by direct inference from downstream gene expression patterns did not indicate significant changes (Figure 4D and Figure S7). However, the functional enrichment analysis on pathway signatures suggested a high activation of p53 pathway at 12 h (Figure S8A,B). This could be related to DNA damage by oxidative stress. An ongoing activation of TP53 has, in other models, been linked to a tipping of the neuronal fate towards death [46].

In order to highlight individual genes with interesting regulation patterns, the extent of gene expression was visualized in various ways (Figure S9–S11). In the time window between 6 and 12 h, several Atf4 and Nrf2-target genes featured among the top 20 up-regulated transcripts. Most notably, AIFM2, also known as FSP1 [47], was the top up-regulated gene, from the 3 h time point onwards. AIFM2 is an NADH oxidoreductase regulated by NRF2, known to localize to biological membranes and lipid droplets [48]. At the cellular membrane, AIFM2 activity prevents ferroptosis by stopping the propagation of lipid peroxides [49]. AIFM2 can also associate with the mitochondrial inner membrane, where it produces NAD^+^ for glycolysis [48].

Cluster analyses of early transcript changes showed that several genes involved in synaptic plasticity were down-regulated at 3 h: synaptotagmin 4 (SYT4), tenascin (TNC) and fibroblast growth factor 18 (FGF18). Another early response (1 h) was the increase in the transcript levels of ribosomal proteins (RPL24, RPL28, RPS3, RPLP0, MRPL36) (Figure S11). This provides evidence for a fast cellular counter-regulation at many levels not directly related to proteasome function.

### 3.5. Early and Transient Perturbation of Neuronal Energy Metabolism after MG Treatment

**Figure 5:**
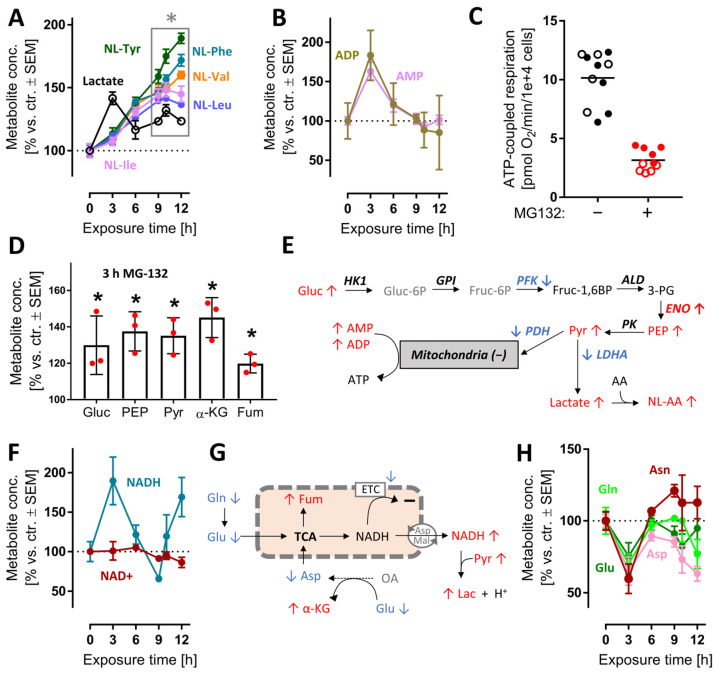
Early perturbation of neuronal energy metabolism after MG-132 treatment. Metabolome and transcriptome samples were obtained as detailed in Figure 1. Data on relative levels of metabolites were extracted from the metabolome data matrix (Figure 1 and Figure S4). (**A**,**B**) Time-dependent changes of lactate, N-lactoyl amino acids (NL-AA), ADP and AMP. (**C**) Data on ATP-coupled respiration were obtained as derivative measure of the overall oxygen consumption rate at 6 h after MG treatment. Filled and empty circles belong to 2 completely separate experiments, each with at least 5 independent replicates. (**D**) Changes of metabolites related to mitochondrial function after 3 h MG. Independent replicates are represented as dots. (**E**) Scheme of glycolysis with mapped measurements for metabolites (3 h) and transcripts (9 h). Up-regulations are shown in red, down-regulations in blue and unchanged levels in black. (**F**) Time-dependent changes of nicotinamides. (**G**) Scheme of mitochondrial metabolism with mapped measurements for metabolites (3 h). (**H**) Time-dependent changes of energy-related amino acids. A complete set of data for the pathways in (**E**) is provided in Figure S12. A full overview of captured metabolites is found in Table S1. A full overview of targeted transcripts is provided in Table S2. Data are means ± SEM of independent replicates. For MG-132 treatments, 3 different samples were analyzed. For controls, 4 replicates were prepared to provide for more robust baseline data. * adjusted-*p* < 0.05; α-KG, α-ketoglutarate; ADP, adenosine diphosphate; AMP, adenosine monophosphate; Fum, fumarate; LDH, lactate dehydrogenase; NAD+, oxidized nicotinamide adenine dinucleotide; NADH, reduced nicotinamide adenine dinucleotide; PDH, pyruvate dehydrogenase; PEP, phosphoenolpyruvate.

After the characterization of overall changes of the metabolome and transcriptome by MG, we wondered whether some of the early metabolite changes could contribute to the massive stress response evident from transcriptome profiling. We focused on energy metabolism, as its changes were already measurable at 3 h (Figure S12) and alterations of its pathways are well-known to be linked to NRF2 and ATF4-like responses [50]. Following proteasome inhibition, lactate rapidly accumulated (peak at 3 h). Increases in lactate point towards increased glycolysis, and possibly to a reduced mitochondrial function. A rise of the cellular lactate concentration was confirmed by the steadily increasing levels of N-lactylated-amino acids, i.e., secondary metabolites formed in cells upon lactate excess (Figure 5A). The ATP metabolites ADP and AMP reached their highest concentrations at 3 h, then gradually returned to control values (Figure 5B). This points towards a drop in cellular energy charge and potentially to an altered mitochondrial function at early time points. To follow up on this, we used Seahorse measurements to study the oxygen consumption rate (OCR) after MG exposure. We found that respiration dropped after 3 h (Figure S12C).

At 6 h, the ATP-coupled respiration was reduced by two thirds (Figure 5C). As overall cellular ATP levels remained constant for at least 6 h (Figure S12D), an increased glycolysis most likely compensated for mitochondrial dysfunction, and possibly led to a renormalization of AMP levels at later time points. In line with this, several glycolysis intermediates (glucose, phosphoenolpyruvate, pyruvate) and some other energy metabolism intermediates (α-ketoglutarate, fumarate) were increased at 3 h. (Figure 5D and Figure S12). The activity of the responsible enzymes was insufficiently captured by the transcriptome approach. Transcripts for some glycolytic enzymes were down-regulated, but the actual catalytic activity cannot be deduced from such data (Figure 5E).

A functioning mitochondrial electron transport chain (ETC) usually maintains high cellular NAD^+^/NADH ratios, while glycolysis tends to decrease the NAD^+^/NADH ratio. At 3 h, NADH accumulated relative to NAD^+^ or control levels (Figure 5F). This suggests that a shift in glycolytic vs. mitochondrial energy metabolism might have taken place. Even with a reduced ETC function, mitochondria may still metabolize glutamine, glutamate and aspartate to NADH, if this is exported to the cytosol [51] (Figure 5H). The marked decrease in these amino acids is well in line with such a metabolic shift, but more data on metabolite fluxes are required to exclude other explanations (Figure 5G). Altogether, the metabolomics data of the 3 h time point suggest rapid and significant shifts in the energy metabolism. This was insofar surprising, as proteasome function is not primarily linked to glycolytic–mitochondrial activity. The rapid effect on so many pathways is likely to have contributed to the stress response. It is also noteworthy that many changes were transient, i.e., the neurons obtained a new homeostatic state, which allowed survival for many more hours.

### 3.6. Late De-Regulation of Pyrimidine Synthesis, Lipid Metabolism and Glutathione Metabolism in MG-Treated Neurons

Interpretation of metabolite changes at late time points is complicated by the fact that they may be (i) a direct, delayed consequence of proteasome inhibition, (ii) a secondary consequence of early changes, (iii) a counter-regulation to halt cell death, (iv) a consequence of cell damage and initiated death processes. Moreover, the intersection of many pathways can lead to high regulation noise on the single metabolite level. Therefore, we focused here only on changes of metabolites that belonged to the significantly regulated (*p* ≤ 0.05) pathways or formed clearly related and correlated groups (Figure S6).

**Figure 6:**
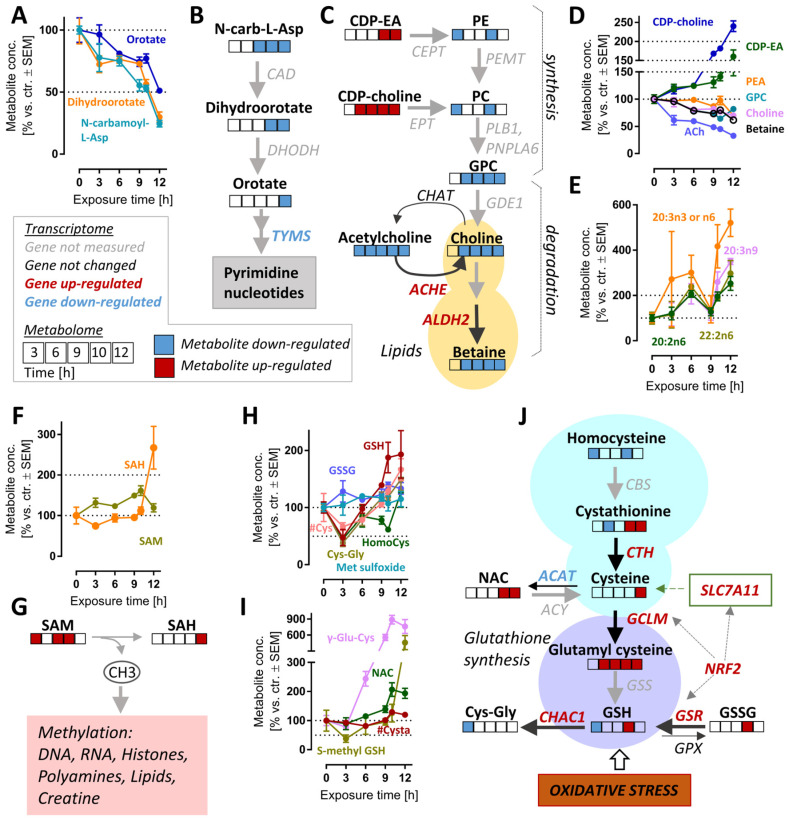
Cell responses relevant to late time points after MG-132 treatment of dopaminergic neurons. Metabolome and transcriptome samples were obtained as detailed in Figure 1. (**A**) Time-dependent changes of pyrimidine de novo synthesis intermediates and (**B**) overview of this pathway with mapped omics responses. (**C**) Choline metabolism pathway diagram and time-dependent changes (**D**) also incorporating the transcriptomic changes of genes coding for relevant enzymes (9 h). (**E**) Levels of polyunsaturated fatty acids (PUFA) over time. (**F**,**G**) Time-dependent changes of the methyl donor S-adenosyl methionine (SAM) and its metabolite S-adenosyl homocysteine (SAH). (**H**,**I**) Time-dependent changes of glutathione-related pathways. (**J**) Graphical overview of GSH metabolism, with mapped omics responses. A complete set of data for these pathways is provided in Figure S8. A full overview of captured metabolites is found in Table S1. A full overview of targeted transcripts is provided in Table S2. Metabolite data are means ± SEM of independent replicates. For MG-132 treatments, 3 different samples were prepared. For controls (DMSO), 4 replicates were prepared to provide for more robust baseline data. #, metabolite quantified by amino acid analyzer; ACAT, acetyl-CoA acetyltransferases; ACh, acetylcholine; ACHE, acetylcholinesterase; ACY, aminoacylase; ALDH2, aldehyde dehydrogenase 2 family member; CAD, carbamoyl-phosphate synthetase 2, aspartate transcarbamylase and dihydroorotase; CBS, cystathionine beta-synthase; CDP-choline, cytidine diphosphate-choline; CDP-EA, cytidine diphosphate-ethanolamine; CEPT, choline/ethanolamine phosphotransferases; CHAT, choline O-acetyltransferase; CTH, cystathionine gamma-lyase; Cys, cysteine; Cysta, cystathionine; DHODH, dihydroorotate dehydrogenase; EPT, CDP-ethanolamine:diacylglycerol ethanolaminephosphotransferase; GCLM, glutamate-cysteine ligase modifier subunit; GDE1, glycerophosphodiester phosphodiesterase 1; GPC, glycerylphosphorylcholine; GPX, glutathione peroxidases; GSH, oxidized glutathione; GSR, glutathione-disulfide reductase; GSS, glutathione synthetase; GSSG, reduced glutathione; HomoCys, homocysteine; Met sulfoxide, methionine sulfoxide; NAC, N-acetyl cysteine; N-carb-L-Asp, N-carbamoyl-L-aspartate; NRF2, NFE2-like BZIP transcription factor 2; PC, phosphatidylcholine; PE, phosphatidylethanolamine; PEMT, phosphatidylethanolamine N-methyltransferase; PLB1, phospholipase B1; PNPLA6, patatin-like phospholipase domain containing 6; SAH, S-adenosylhomocysteine; SAM, S-adenosylmethionine; SLC7A11, cystine/glutamate transporter; TYMS, thymidylate synthetase.

The pyrimidine de novo synthesis pathway was significantly altered after >10.5 h (Figure S6B). The down-regulated metabolites within this pathway included orotate, dihydroorotate and N-carbamoyl-aspartate. These intermediates were depleted by >2-fold (levels of <50%) by 12 h (Figure 6A,B). In agreement with these changes, the expression of thymidylate synthase (TYMS), an enzyme which catalyzes a rate-limiting step in thymidine synthesis, was down-regulated (Supplementary Table S2). The reduction in pyrimidine synthesis may indicate a general attenuation of biosynthetic processes. This is consistent with the observed ATF4 stress response (Figure 4D and Figure S7).

In metabolomics studies of cell death models, decreasing metabolite levels might be a technical artefact of normalization or an indirect (unspecific) consequence of cell lysis. Such concerns are of relatively low relevance here, as we also observed significant up-regulations of transcripts and metabolites. For instance, the lipidomic landscape showed complex up- and down-regulations. Some key precursors of phospholipid synthesis (CDP-choline, CDP-ethanolamine) were clearly up-regulated (at 6–12 h), while phospholipid synthesis and glycerophospholipid metabolism were significantly up-/down-regulated at 6–12 h (Figure S6B). While there were many of the long-chain fatty acids that were strongly up-regulated (Figure 6E), choline metabolites (ACh, choline and betaine) also showed a highly-correlated depletion (Figure 6C,D).

The synthesis of choline (and related metabolites) requires several S-adenosylmethionine (SAM)-dependent methylation reactions. We enquired whether the strong and consistent decrease in choline was explained by SAM depletion. However, the levels of this important methyl donor were rather increased, and S-adenosyl homocysteine (SAH), a metabolite of SAM, more than doubled by 12 h. We concluded that cellular methylation capacity was maintained for at least 12 h after proteasome inhibition, and depletion of choline is likely to be due to other reasons (Figure 6F,G).

A particularly interesting set of metabolites up-regulated at late time points are small molecular weight cellular thiols such as GSH, cysteinyl-glycine (Cys-Gly) and γ-glutamyl-cysteine (γ-Glu-Cys) (Figure S3C). We, therefore, took a closer look at GSH-related pathways (Figure 6H,I and Figure S13C,D). Besides GSH, N-acetyl cysteine (NAC), S-methyl GSH and homocysteine also increased two-fold at 12 h compared to the control. At first glance, it might look counter-intuitive that GSH levels are increased, although proteasome inhibition causes oxidative stress [24], clearly indicated here by NRF2 and ATF4 responses. Upon closer inspection, Cys, Cys-Gly, homocysteine, GSH and S-methyl GSH are indeed initially depleted (3 h), then the stress response up-regulates genes of GSH metabolism (SLCA11, GCLM, GSR), and also strongly increased expression of genes involved in the GSH reduction pathway (ME1, TIGAR, GPX2).

On a pathway level, several of the metabolite changes look heterogeneous, as most metabolites belong to several pathways, some of which might lead to contrasting effects on metabolite pools (Figure 6J). Several pathways contribute to GSH synthesis and might be responsible for its rise (e.g., the trans-sulfuration pathway, involving cystathionine). At the same time, ongoing oxidative stress affects some metabolites. Moreover, some of the degradation pathways (e.g., driven by Chac1) are also strongly up-regulated at transcriptome level.

Given this general difficulty of metabolomics analysis, some of the observed regulations (early alterations in energy metabolism and late counter-regulation of oxidative stress) give a strikingly consistent picture and indicate that cells adapted their homeostasis at many levels for up to 12 h. The path towards cell death was finally decided only after this period (Figure S14). Co-treatment with an iron chelator did not rescue the cells, suggesting that ferroptosis is not the modality of death (Figure S15).

## 4. Conclusions

Our study revealed that the neuronal metabolic response to proteasome inhibition is very fast (≤3 h) and highly dynamic over time, whereas mRNA levels showed a slower and predominantly monotonic time course profile, with most changes between 6 and 12 h MG treatment.

The highest level of metabolic imbalance was observed at 3 h of MG treatment. The most conspicuous changes were related to the energy metabolism and the GSH-antioxidant defense. Since no metabolomics data were obtained before the 3 h time point, earlier sampling times are suggested by these findings for all follow-up studies.

Most of the delayed metabolite changes can be interpreted as successful counter-regulations of the initial stress. Most notable is the strong up-regulation of GSH, the most important redox buffer of the cells. A similar GSH counter-regulation was also observed in hepatocytes treated with the antibiotic nitrofurantoin [52] and in neurons exposed to the mitochondrial toxicant MPP+ [50], or to the drug methamphetamine ± CEP1347 [53]. Some other changes (altered levels of small amino acids) might be interpreted as adaptations to provide precursors for GSH synthesis. These findings confirm metabolomic profiling as a powerful tool for identifying patterns of toxicant-induced metabolic shifts [54,55]. Another advantage of this technology over individual metabolite measurements proved to be the higher sensitivity for changes and the easier interpretation of correlated effects of several metabolites. Some examples suggest that by combining two omics methods, the emerging patterns can be better put into biological context [32,50,56]. For instance, the earliest gene markers of oxidative stress were up-regulated already at 3 h (AIFM2, NQO1, GCLM, SQSTM1, ATF4). At 6 h, the transcriptomic response clearly reflected the adaptations to proteasome inhibition, i.e., increased expression of proteasome subunits, chaperones (HSF1 pathway) and redox-related proteins (NRF2 and ATF4 pathways). Altogether, these results suggest concerted attempts made by the neurons to overcome the negative consequences of the proteasome block, and of early energetic and oxidative stress.

Two molecular targets with potential for drug intervention also emerged: the NAD(H)-dependent oxidoreductases AIFM2 (FSP1) and malic enzyme (ME1). These two showed the most significant transcriptional up-regulation throughout the time course. The many roles of FSP1 can be categorized as pro-survival (prevention of ferroptosis by reducing lipid peroxides [47], mitochondrial uncoupling [48]) and pro-death (induction of apoptosis [57]). Given its multifaceted functions, FSP1 could be an underestimated cell fate regulator/switch. We hypothesize that MG-induced early activation of FSP1 might be important for initial cell survival despite oxidative stress, thus allowing cells the time to repair. Since no studies have so far linked proteasome inhibition to FSP1 induction, follow-up experiments will be needed to clarify the role of the observed drastic regulation in cell death. The role of ME1 also needs further investigation. It is a known regulator of energy metabolism, and the reaction catalyzed by the enzyme generates NADPH, which is required to fight oxidative stress [58]. Thus, its regulation is consistent with the oxidative stress observed here.

This study has several limitations and gaps. For instance, the direct molecular link between proteasome inhibition and oxidative stress could not be clarified, and further studies are required. Additionally, it remains unclear whether there is a single event or metabolite that is eventually responsible for cell death.

Finally, our results highlight the importance of sampling time and show how easily the cellular response can be missed or misinterpreted if a toxicant exposure study is limited to only a few time points. For our study, some additional early time points would have been beneficial. Possibly, late time points beyond the point-of-no-return may also have been informative. We showed that neurons can be rescued from MG-132 by the addition of Cys (Figure S14) or GSH [24] at time points up to 10 h after the start of the experiment. However, our data suggest that the adaptive changes (i.e., the protective counter-regulations of cell metabolism, such as GSH up-regulation) continue (on metabolome and transcriptome levels) until 12 h. Possibly protective and detrimental regulations run in parallel for a while, and having data from more than one time point of this phase would be of interest. For instance, it would be interesting if the strength and pattern (target gene panel) of p53 pathway activation changes at times beyond 12 h.

Even though the exact pathway or ensemble of metabolites leading to cell death is as yet unclear, the model system established here may allow to define key switches, pathways and regulations susceptible to neuroprotective drugs.

## Data Availability

The data presented in this study are available in Supplementary Tables S1 and S2.

## Supplementary Materials

The following supporting information can be downloaded at: https://www.mdpi.com/article/10.3390/antiox12010164/s1, Supplementary Figures S1–S15: Supplementary figure; Supplementary Tables S1 and S2: Metabolomics and transcriptomics statistical results.

## Abbreviations

AA	amino acid(s)
AA-Ac	acetylated amino acids
AIFM2	apoptosis-inducing factor mitochondria associated 2
ATF4	activating transcription factor 4
ATP	adenosine triphosphate
cAMP	N6,2′-O-dibutyryladenosine 3′,5′-cyclic monophosphate
DEG	differentially expressed gene
DGE	differential gene expression
ETC	electron transport chain
GDNF	glial-derived neurotrophic factor
GSEA/MSEA	gene/metabolite set enrichment analysis
GSH	reduced glutathione
MG	proteasome inhibitor MG-132
NAD(H)	nicotinamide adenine dinucleotide (NAD+: oxidized, NADH: reduced)
NAT	N-terminal acetyltransferases
NRF2	NFE2-like BZIP transcription factor 2 (NFE2L2)
ORA	overrepresentation analysis
PCA	principal component analysis
PD	Parkinson’s disease
Q	ubiquinone or coenzyme Q
ROS	reactive oxygen species
TCA	citric acid cycle or Krebs cycle or tricarboxylic acid cycle
TF	transcription factor(s)
Ubi	ubiquitin
Ubi-Prot	ubiquitinated protein(s)
