# Supplementary material for: Dynamic Metabolic and Transcriptional Responses of Proteasome-Inhibited Neurons
Source: Antioxidants (Basel). 2023 Jan 10;12(1):164. doi: 10.3390/antiox12010164 (PMC9854434; doi:10.3390/antiox12010164)
Supplement: Supplementary file 1 [file antioxidants-12-00164-s001.zip › antioxidants-2117752 Suppl Fig Final S1-15.pdf]

Supplementary Material

Dynamic metabolic and transcriptional responses of proteasome-inhibited neurons

Ilinca Suciu [1,2], Johannes Delp [1], Simon Gutbier [1], Anna-Katharina Ückert [1,3], Anna-Sophie Spreng [1,2], Philipp Eberhard [4], Christiaan Karreman [1], Falk Schreiber [4,5], Katrin Madjar [6], Jörg Rahnenführer [6], Ivana Celardo [1], Ivano Amelio [7] and Marcel Leist [1,8,*]

Table of Contents

Element	Page	Title
Fig. S1	2	**Typical downstream consequences of proteasome inhibition in LUHMES cells treated with MG-132**
Fig. S2	3	**Overview of all metabolome changes over time**
Fig. S3	4	**Comparison between early and late metabolic perturbations**
Fig. S4	5	**Correlated down-regulation of metabolites over time during early or late time points**
Fig. S5	6	**Correlated accumulation of metabolites over time during early or late time points**
Fig. S6	7	**Changes of metabolic pathways over time**
Fig. S7	8	**Activity predictions for DoRothEA master regulators**
Fig. S8	9	**Activity predictions for progeny pathways**
Fig. S9	10	**Display of genes most strongly regulated by MG-132 exposure**
Fig. S10	11	**Overview of significantly dysregulated genes**
Fig. S11	12	**Integrated view of the most highly dysregulated genes over time**
Fig. S12	13	**Concentrations of all measured metabolites related to glycolysis and the citric acid cycle**
Fig. S13	14	**Change of oxidative stress- and lipid-related metabolites over time**
Fig. S14	15	**Rescue time window of proteasome-inhibited LUHMES cells by using a thiol supply**
Fig. S15	16	**Effect of iron chelation on MG-132-induced neuronal cell death**

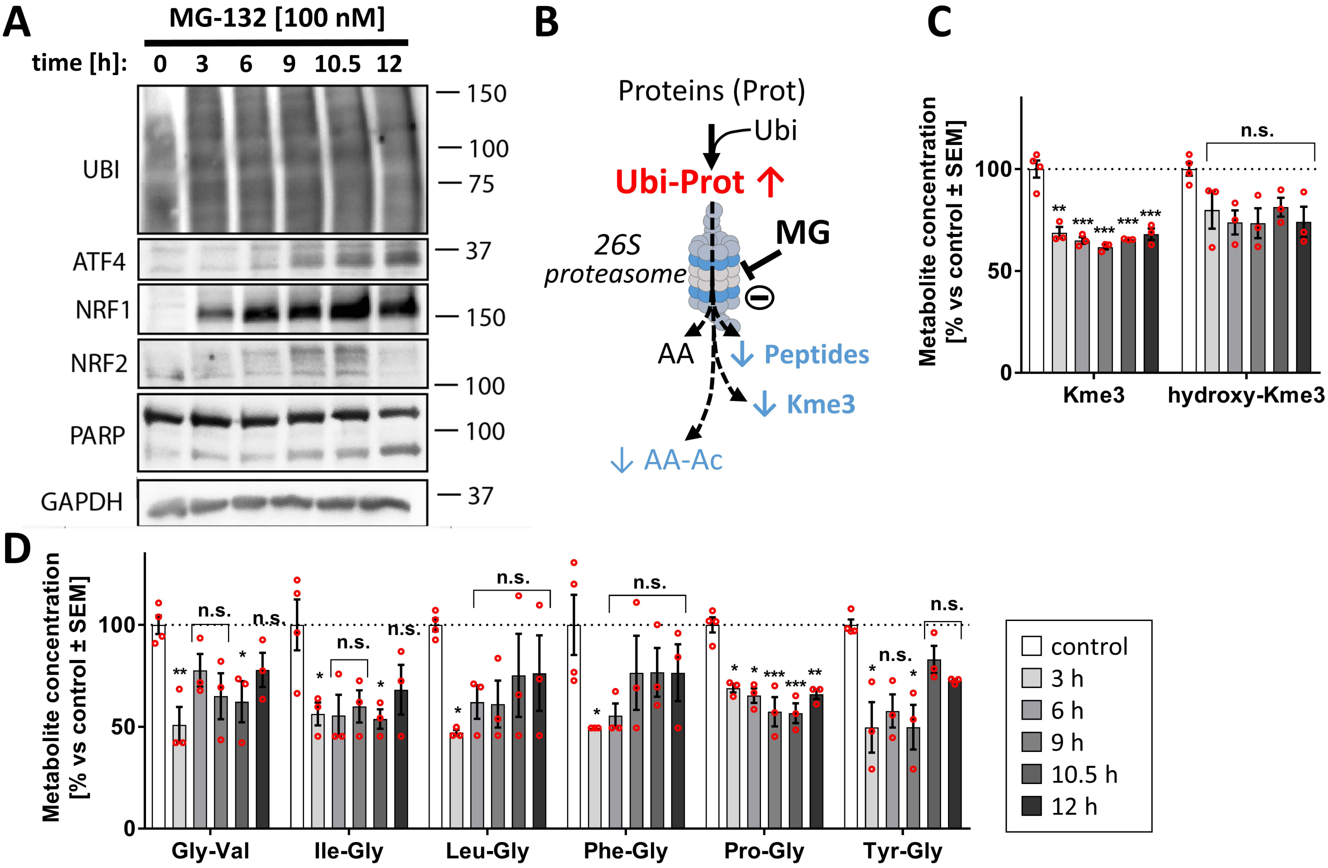

Fig. S1: Typical downstream consequences of proteasome inhibition in LUHMES cells treated with MG-132

(**A**) For the metabolomics experiment, parallel LUHMES samples treated with 100 nM MG-132 (MG) were prepared for assay validation. Previously shown responses (Gutbier et al. 2018) to proteasome inhibition were confirmed by Western Blot: accumulation of ubiquitinated proteins, and induction of ATF4, NRF1 and NRF2 stress response pathways. (**B**) Scheme showing the predicted effect of MG on proteostasis. The ubiquitination of proteins (Ubi-Prot) normally leads to their degradation by the 26S proteasome. Inhibition of the proteasome causes an accumulation of Ubi-Prot. In parallel, the downstream products of degradation are depleted: amino acids (AA) and small peptides. While amino acids are part of many metabolic pathways unrelated to proteasome activity, small peptides (here – dipeptides) are mainly generated by proteasomal activity. Quantification of (**C**) trimethylated Lys and (**D**) dipeptides over the time course of MG treatment. Metabolite data are means ± SEM of independent replicates. For MG-132 treatments, 3 different samples were analyzed. For controls (DMSO), 4 replicates were prepared to provide for more robust baseline data. * *p*-value ≤ 0.05; ** *p*-value ≤ 0.01; ** *p*-value ≤ 0.001; n.s., not significant; AA, amino acids; Kme3, N6,N6,N6-trimethyllysine; Ubi, ubiquitin; Ubi-Prot, ubiquitinated proteins.

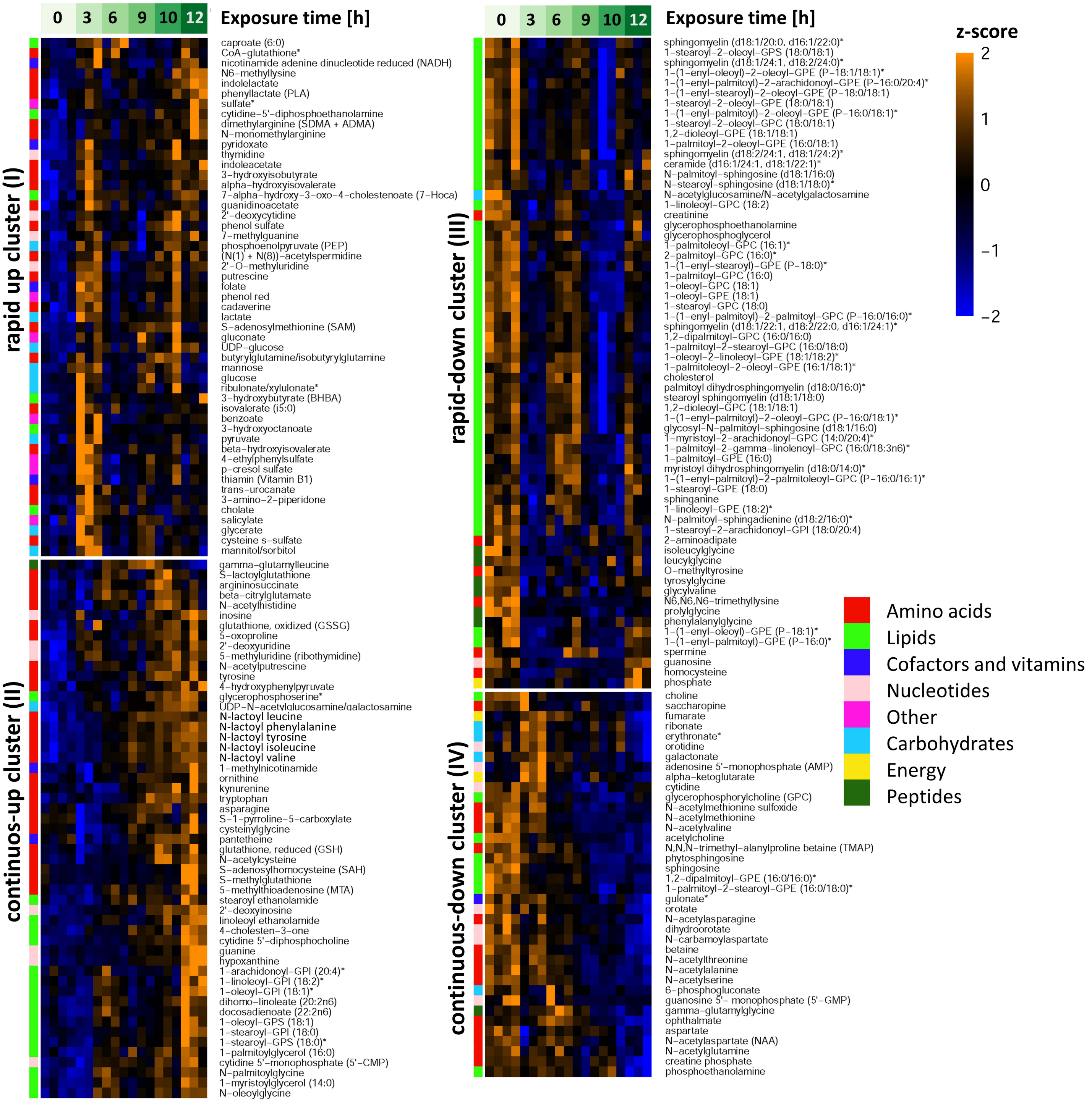

Fig. S2: Overview of all metabolome changes over time.

Alltogether, 386 metabolites were quantifiable in control samples (Suppl. Table 1). Based on the limma statistics, 206 of these metabolites were significantly changed ($p \leq 0.05$) at least at one time point by MG-132 treatment. Unsupervised clustering of metabolite time profiles using the hierarchical clustering method. Heatmap representation of the clustered metabolites (rows) at the tested time points (columns). Their corresponding trendlines are shown in Fig. 3C. The heat map colors represent z-scores of the row-wise normalized expression values for each metabolite. All samples analyzed in this study were prepared separately and treated as independent (biological replicates). For the metabolomics analysis, 3 treated samples per time point, and 4 control samples were used. A color code was used to visualize the major biochemical groupings (e.g. lipids, nucleotides) of the metabolites.

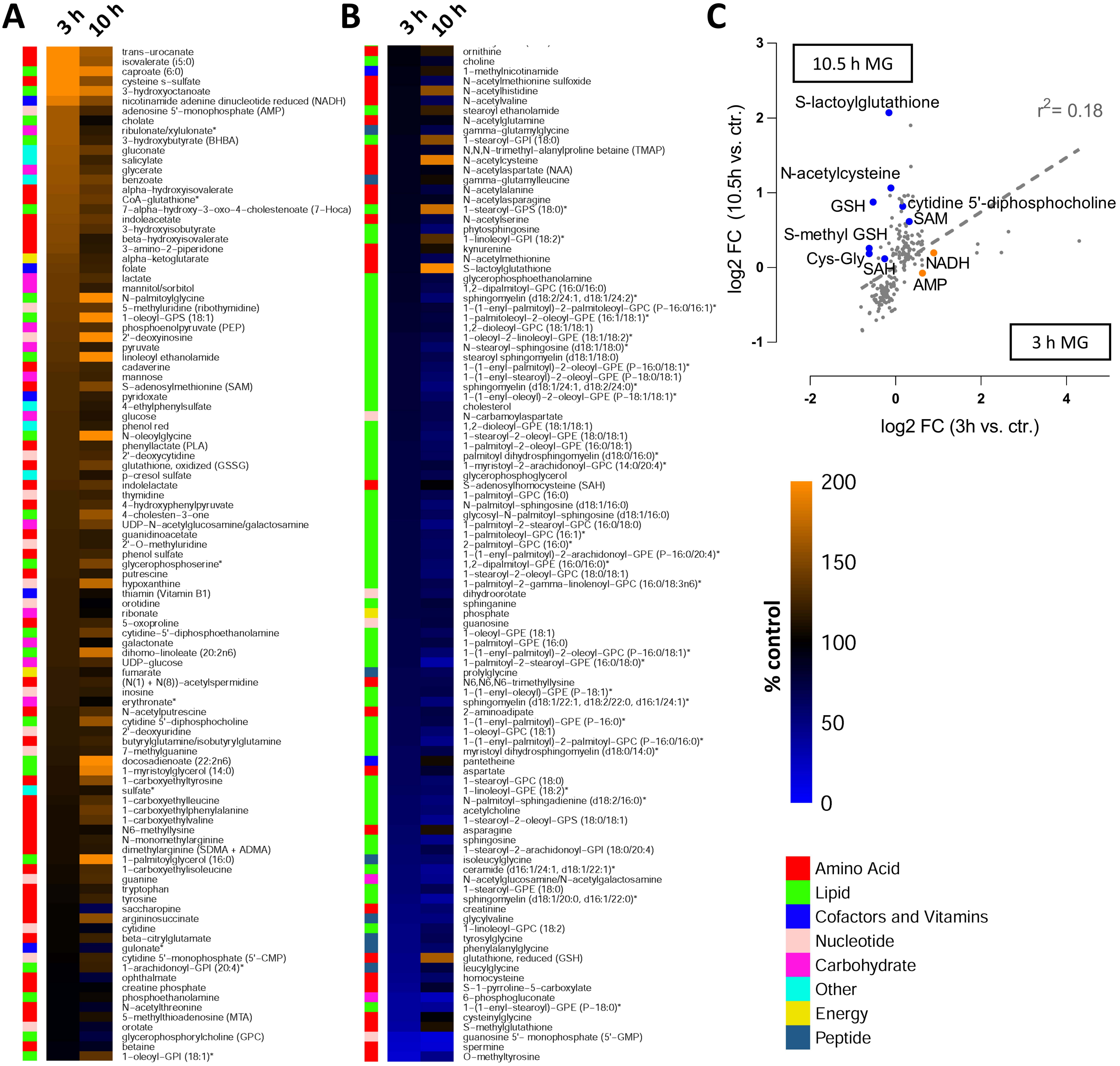

Fig. S3: Comparison between early and late metabolic perturbations

(**A**), (**B**) Metabolite concentration levels at 3 h and 10.5 h relative to the DMSO control are shown side-by-side in a heat map. Data are not z-scores as in Fig. S2. (**A**) Nearly all metabolites that are upregulated at 3 h are also upregulated at 10.5 h. (**B**) Nearly all metabolites that are depleted at 3 h are downregulated at 10.5 h. (**C**) A scatter plot was used to compare the responses at 3 h [x] and 10.5 h [y], both given as log2 fold changes relative to the DMSO control. The color-coded dots represent some exemplary relative up-/down-regulations.

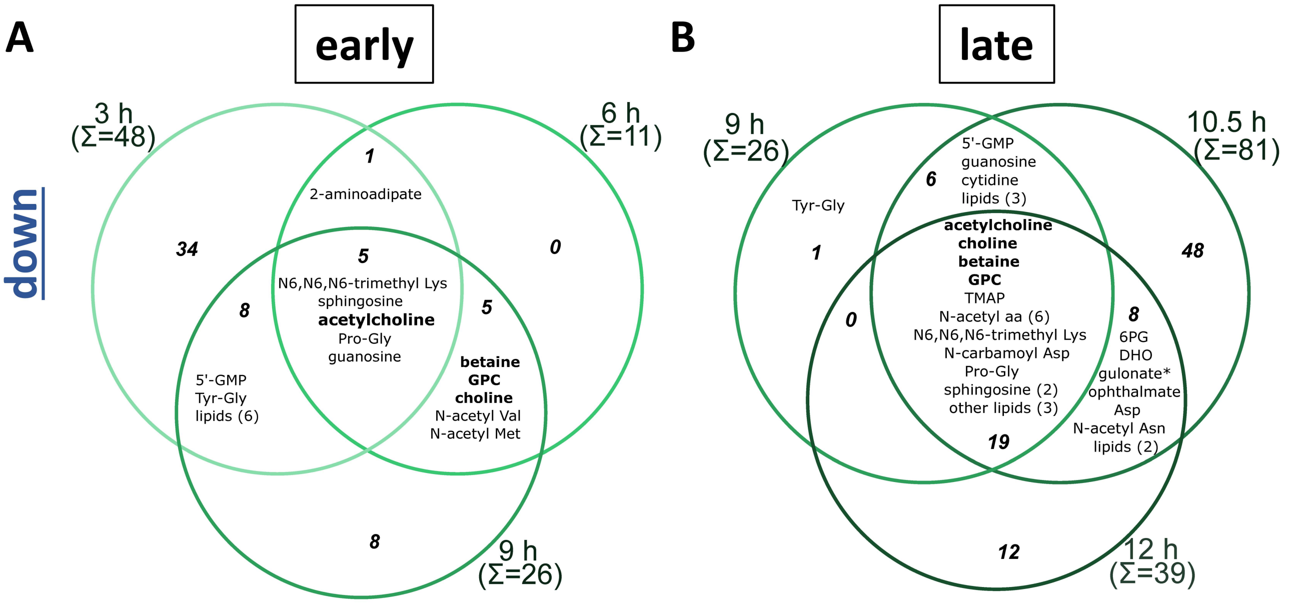

Fig. S4: Correlated down-regulation of metabolites over time during early or late time points.

To understand if the downward metabolic changes are correlated over time, a comparison between the significantly depleted metabolites at different time points was conducted. Venn diagrams were used to check the relationship amongst early-intermediate responses, and intermediate-late responses to the MG-132 treatment. Numbers in the diagram indicate the number of metabolites falling into the respective areas. (**A**) Intersection of the early-intermediate downregulated metabolites (3 h, 6 h and 9 h). (**B**) Intersection of the intermediate-late downregulated metabolites (9 h, 10.5 h and 12 h). The common elements are listed in the area of overlap.

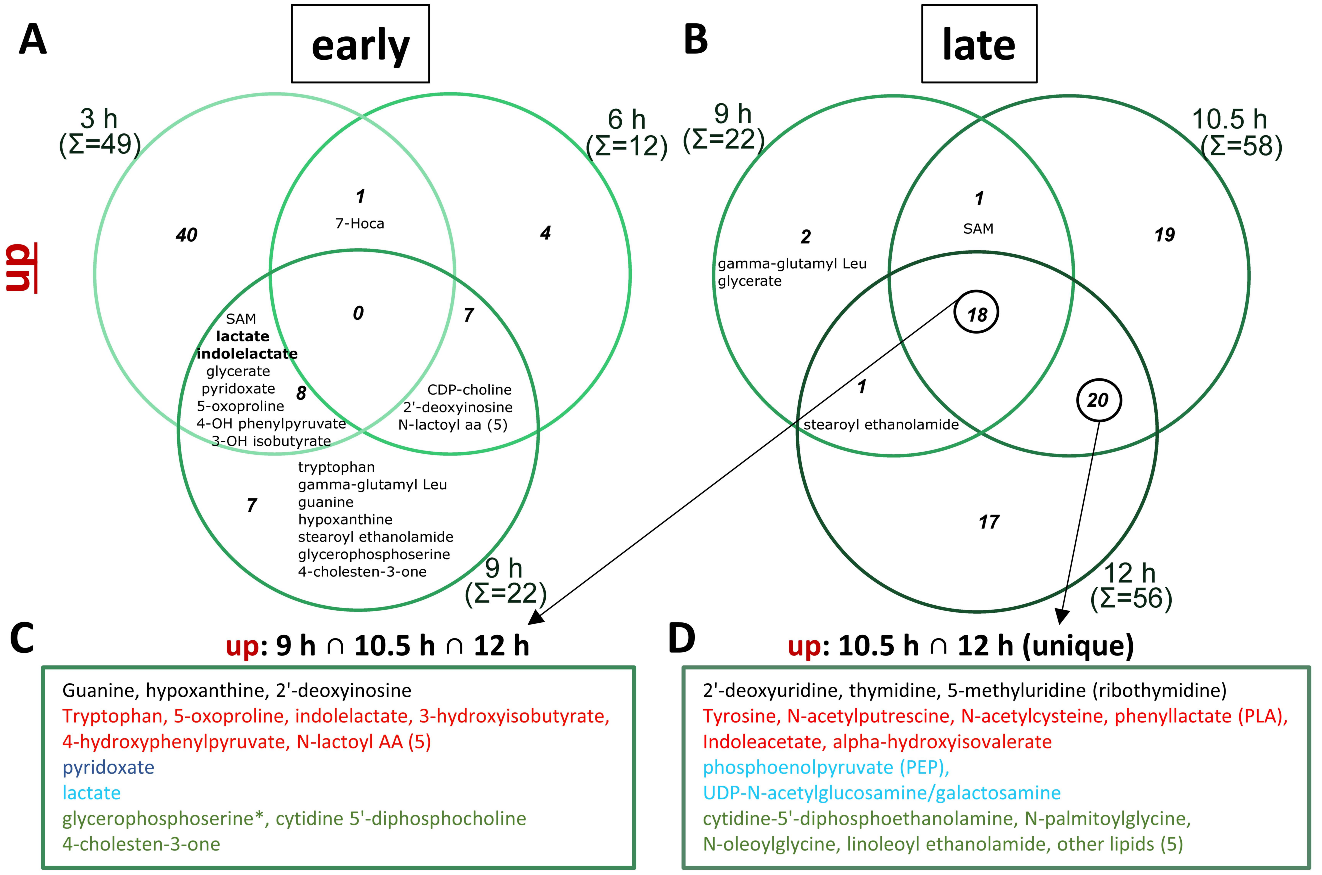

Fig. S5: Correlated accumulation of metabolites over time during early or late time points.

To understand if the upward metabolic changes are correlated over time, a comparison between the significantly accumulating metabolites at different time points was conducted. Venn diagrams were used to check the relationship between on the one hand early-intermediate responses, and on the other hand intermediate-late responses to the MG-132 treatment. Numbers in the diagram indicate the number of metabolites falling into the respective areas. (**A**) Intersection of the early-intermediate upregulated metabolites (3 h, 6 h and 9 h). (**B**) Intersection of the intermediate-late upregulated metabolites (9 h, 10.5 h and 12 h). Where the space allowed, the common elements were listed in the area of overlap. The metabolites significantly upregulated at all 3 later time points are listed in (**C**). The metabolites significantly upregulated at all 10.5 and 12 h but not at 9 h are listed in (**D**).

A

Pathway	time [h] 3	6	9	10.5	12	Ref.	ID
Pentose phosphate pathway	0.01	*n.s.*	*n.s.*	*n.s.*	*n.s.*	K	hsa00030
cAMP signaling pathway	0.01	*n.s.*	*n.s.*	*n.s.*	*n.s.*	K	hsa04024
HIF-1 signaling pathway	0.01	*n.s.*	*n.s.*	*n.s.*	*n.s.*	K	hsa04066
GABAergic synapse	0.01	*n.s.*	*n.s.*	*n.s.*	*n.s.*	K	hsa04727
Glucose-alanine cycle	0.02	*n.s.*	*n.s.*	*n.s.*	*n.s.*	S	SMP00127
Calcium signaling pathway	0.03	*n.s.*	*n.s.*	*n.s.*	*n.s.*	K	hsa04020
Urea cycle	0.03	*n.s.*	*n.s.*	*n.s.*	*n.s.*	S	SMP00059
Glucagon signaling pathway	0.03	*n.s.*	*n.s.*	*n.s.*	*n.s.*	K	hsa04922
Glycolysis / Gluconeogenesis	0.04	*n.s.*	*n.s.*	*n.s.*	*n.s.*	K	hsa00010
Malate-aspartate shuttle	0.05	*n.s.*	*n.s.*	*n.s.*	*n.s.*	S	SMP0000129
Ammonia recycling	0.05	*n.s.*	*n.s.*	*n.s.*	*n.s.*	S	SMP0000009
Gluconeogenesis	*n.s.*	*n.s.*	*n.s.*	*n.s.*	*n.s.*	S	SMP00128

B

Pathway	time [h] 3	6	9	10.5	12	Ref.	ID
Phospholipid biosynthesis	*n.s.*	0.01	0.004	0.04	*n.s.*	S	SMP0000025
Choline metabolism in cancer	*n.s.*	0.03	*n.s.*	*n.s.*	*n.s.*	K	hsa05231
Glycerophospholipid metabolism	*n.s.*	*n.s.*	0.01	0.02	0.02	K	hsa00564
Ether lipid metabolism	*n.s.*	*n.s.*	0.03	0.01	*n.s.*	K	hsa00565
Betaine metabolism	*n.s.*	*n.s.*	0.05	*n.s.*	*n.s.*	S	SMP0000123
Dopaminergic synapse	*n.s.*	*n.s.*	0.03	*n.s.*	*n.s.*	K	hsa04728
Serotonergic synapse	*n.s.*	*n.s.*	0.03	*n.s.*	*n.s.*	K	hsa04726
Pyrimidine metabolism	*n.s.*	*n.s.*	*n.s.*	0.04	0.02	K	hsa00240
Phosphatidylcholine biosynthesis	*n.s.*	*n.s.*	*n.s.*	*n.s.*	0.003	S	SMP14287
Phosphatidylethanolamine biosynthesis	*n.s.*	*n.s.*	*n.s.*	*n.s.*	0.01	S	SMP15109

Fig. S6: Changes of metabolic pathways over time.

Pathway enrichment analysis was performed using 2 different methods: metabolite set enrichment analysis (MSEA) based on KEGG pathways (K) and overrepresentation analysis (ORA) based on SMPD pathways (S). Both MSEA and ORA give the same weight to all metabolites in the pathway and don‘t take into account the relationship between these elements. The type of algorithm and database used are indicated in the reference (Ref.) column. The pathway identifiers are specified in the ID column. (**A**) Table of early deregulated pathways and their p-value at all tested exposure times. (**B**) Table of pathways deregulated at any of the later time points (6 – 12 h) and their *p*-value at all tested exposure times. The *p*-values indicate the likelihood of the respective pathway not being regulated. *n.s.*: not significant.

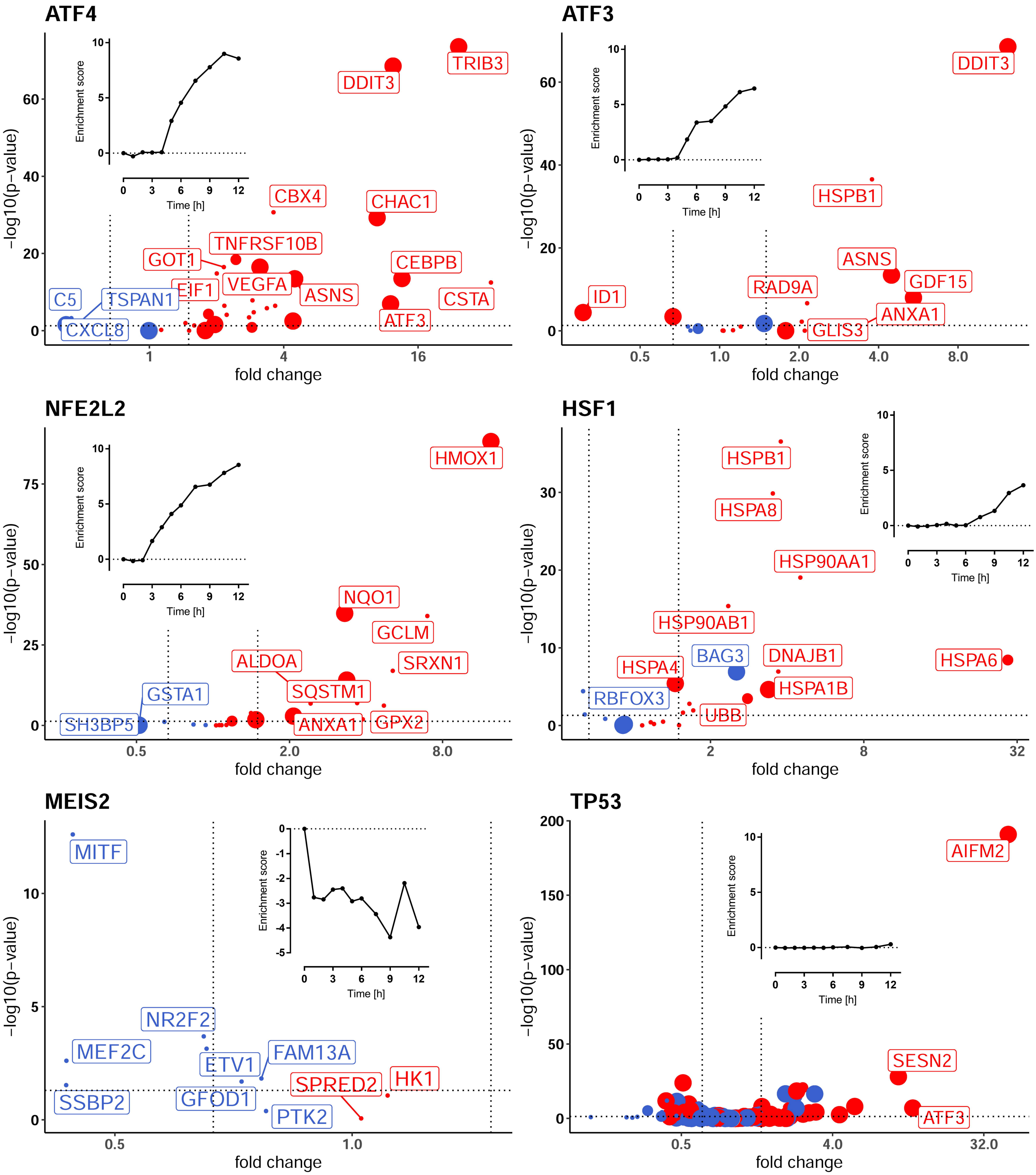

Fig. S7: activity predictions for DoRothEA master regulators

The activity of master regulators was predicted based on the expression of their targets using manually curated DoRothEA human regulons (Garcia-Alonso et al. 2019) with confidence levels A, B and C which denote high supportive evidence for the transcription factor-target relationship. Correlated enrichment scores were calculated on the Wald statistic (stat) using the weighted mean for all experimental conditions. The log2 fold change values of the genes used for the pathway activity predictions were plotted against their p-values. Volcano plots show the target genes of transcription factors ATF4, ATF3, NRF2/NFE2L2, HSF1, MEIS2 and TP53. The sign of the product between a target's mode of regulation and stat can be positive (red) or negative (blue). The inserts show the enrichment scores over time, from 0 until 12 h after the MG treatment.

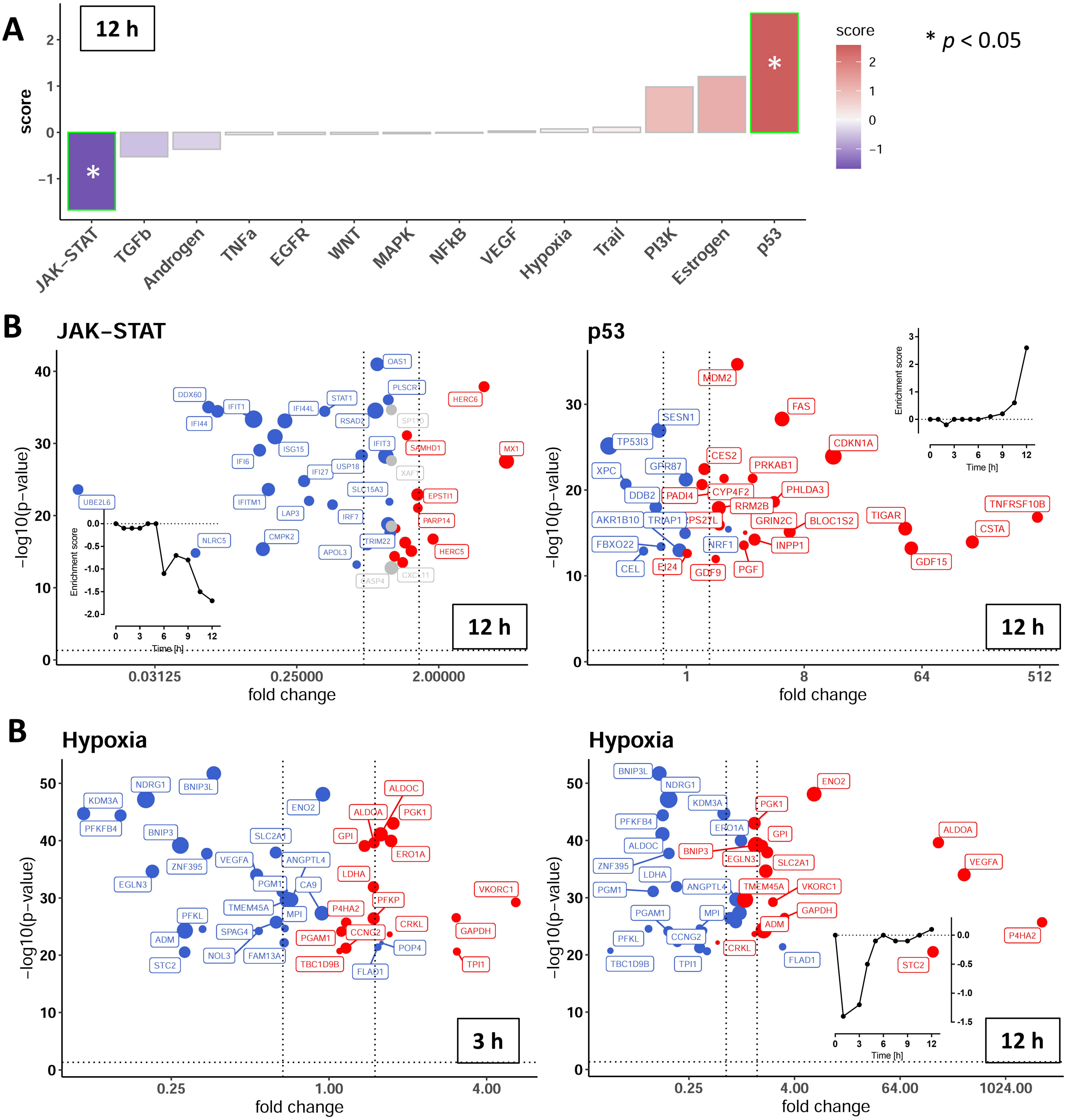

Fig. S8: activity predictions for progeny pathways

The activity of master regulators was predicted based on the expression of their targets using manually curated progeny human pathways. For each experimental condition, correlated enrichment scores were calculated by applying the weighted mean (WMEAN) method to the Wald statistic (stat). (**A**) Enrichment scores of all progeny pathways at 12 h MG. (**B**),(**C**) The log2 fold change values of the genes used for the pathway activity predictions were plotted against their p-values. (**B**) Volcano plots showing the genes involved in JAK-STAT and p53 signaling after 12 h MG. (**C**) For hypoxia, two separate volcano plots were generated, one for 3 h (left) and the other for 12 h (right). The sign of the product between a target's mode of regulation and stat can be positive (red) or negative (blue). The inserts show the enrichment scores over time, from 0 to 12 h MG treatment.

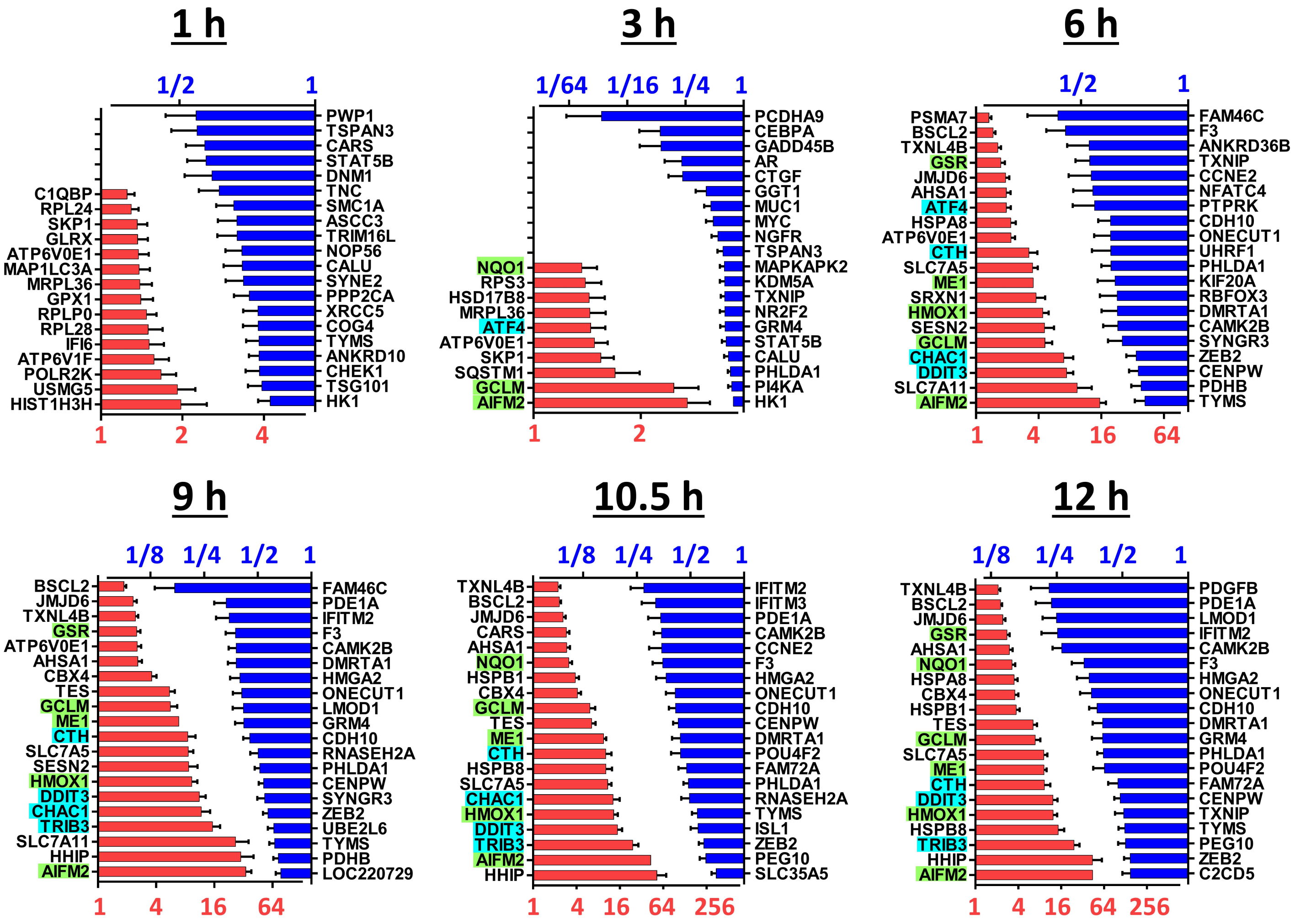

[x-scale: FC compared to 0 h]

Fig. S9: Display of genes most strongly regulated by MG-132 exposure

Following the differential gene expression analysis (Wald statistics) of each test condition against the DMSO control (Suppl. Table 2), for each of the selected time points, the significantly dysregulated genes (adjusted p-value ≤ 0.05) were ranked according to their fold changes. Bar plots show the top 20 up- (red) and respectively downregulated (blue) genes, with separate axes for up- (bottom) and down-regulated (top) genes (in fold changes (FC)). Few genes (NQO1, HMOX1, AIFM2, ME1, GSR, GCLM) related to oxidative stress are highlighted (green) for an easier overview. The genes TRIB3, CHAC1, DDIT3, ATF4 (cell damage indicators) were highlighted in turquoise. All samples analyzed in this study were prepared separately and treated as independent (biological replicates). For the transcriptomics analysis, 6 samples were produced and measured for each condition.

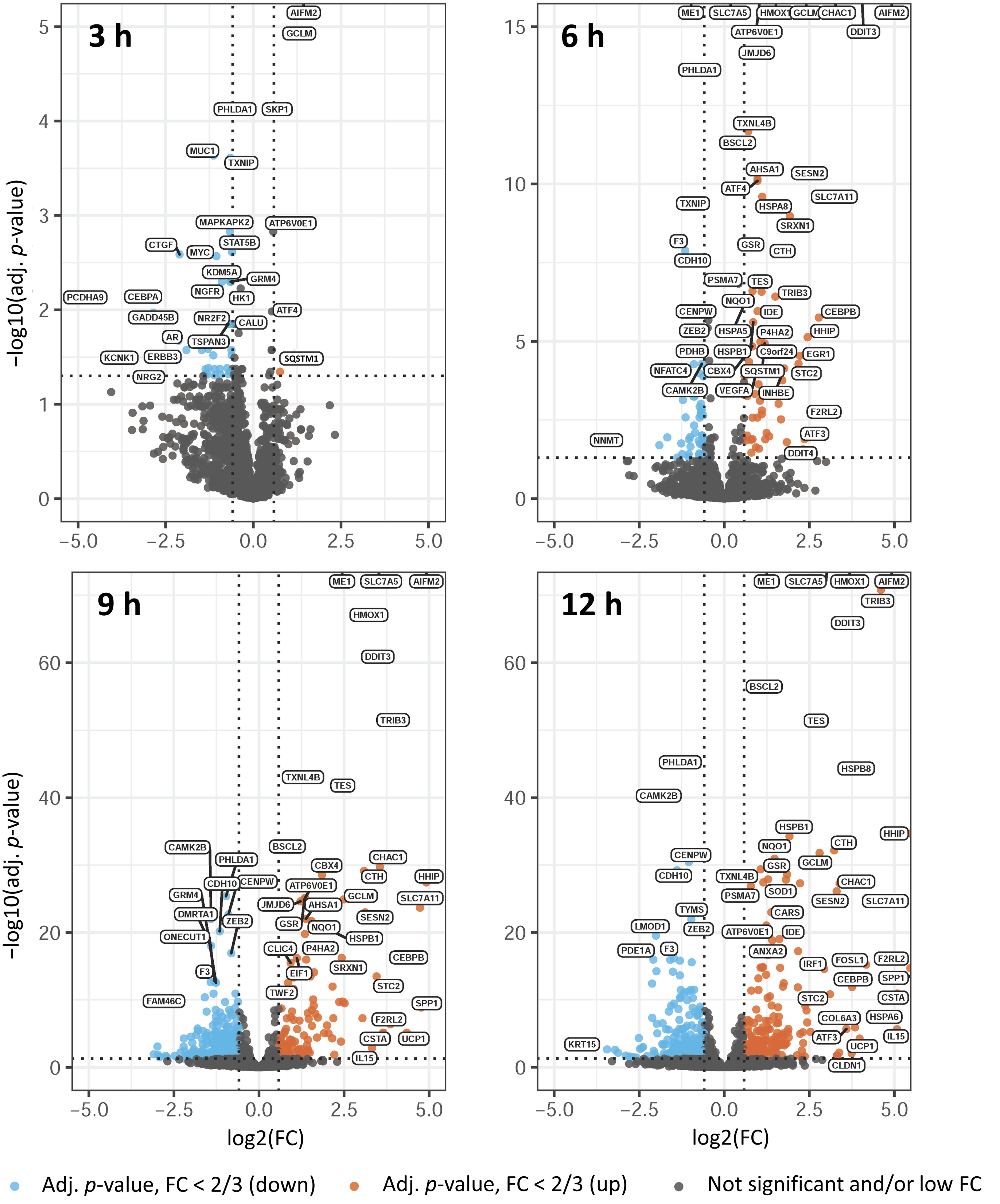

Fig. S10: Overview of significantly dysregulated genes.

The differential gene expression data was explored in Volcano plots to illustrate the relationship between log10 adjusted *p*-value and log2 fold change (FC) after 3, 6, 9 and 12 h exposure of LUHMES cells to MG-132. Some of the most responsive genes are highlighted.

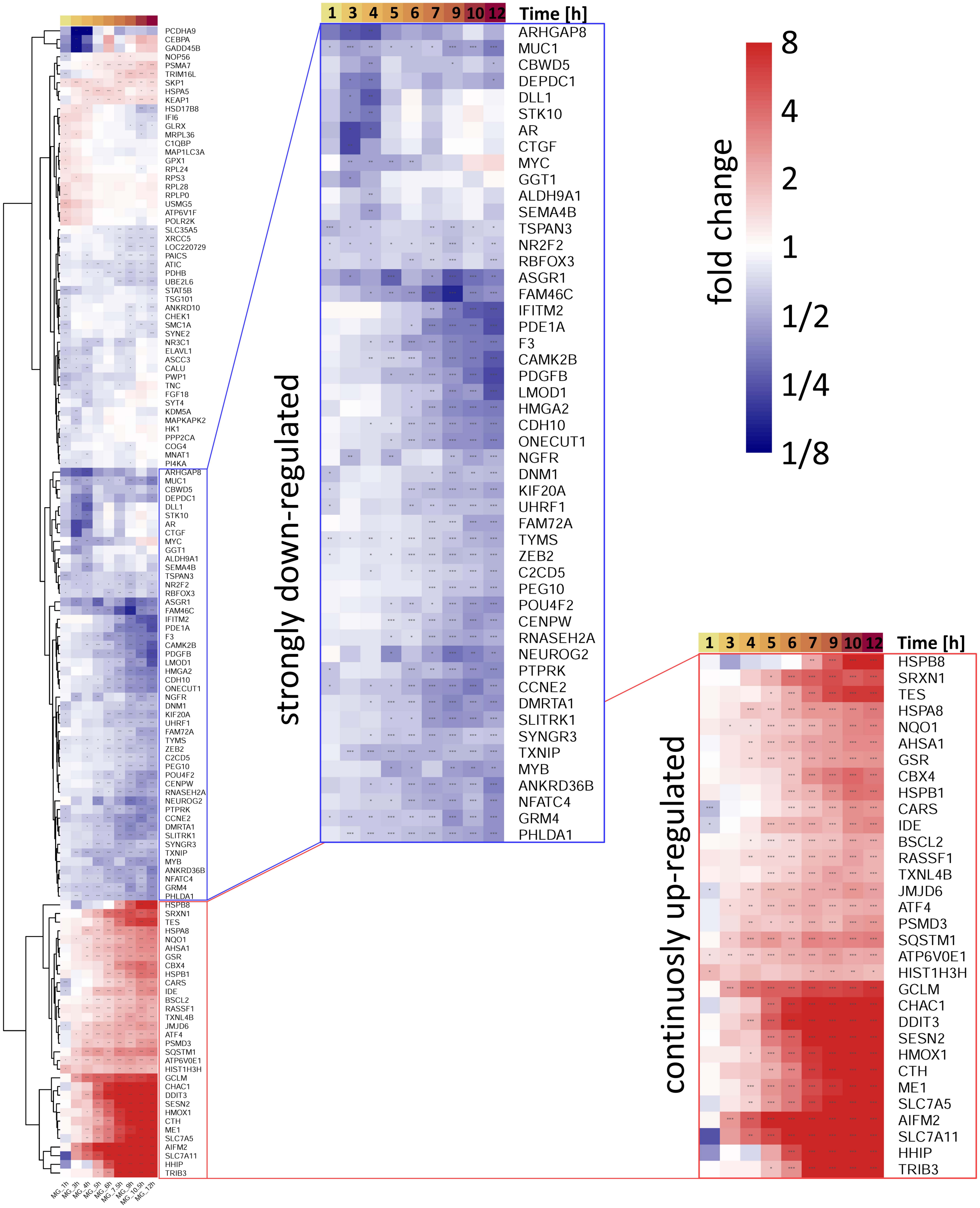

Fig. S11: Integrated view of the most highly dysregulated genes over time

All hits from Fig. S9 were collected in a list which was used to extract (from the gene expression matrix) and combine the statistical information of these genes for all exposure times. The obtained log2 fold change matrix was then clustered by rows (i.e. genes) using the hierarchical clustering method. Heat map of the clustered hits, where a linear blue-white-red color scale was used for fold-changes and p-adjusted values were represented by asterisks. * adjusted p-value ≤ 0.05; ** adjusted p-value ≤ 0.01; *** adjusted p-value ≤ 0.001.

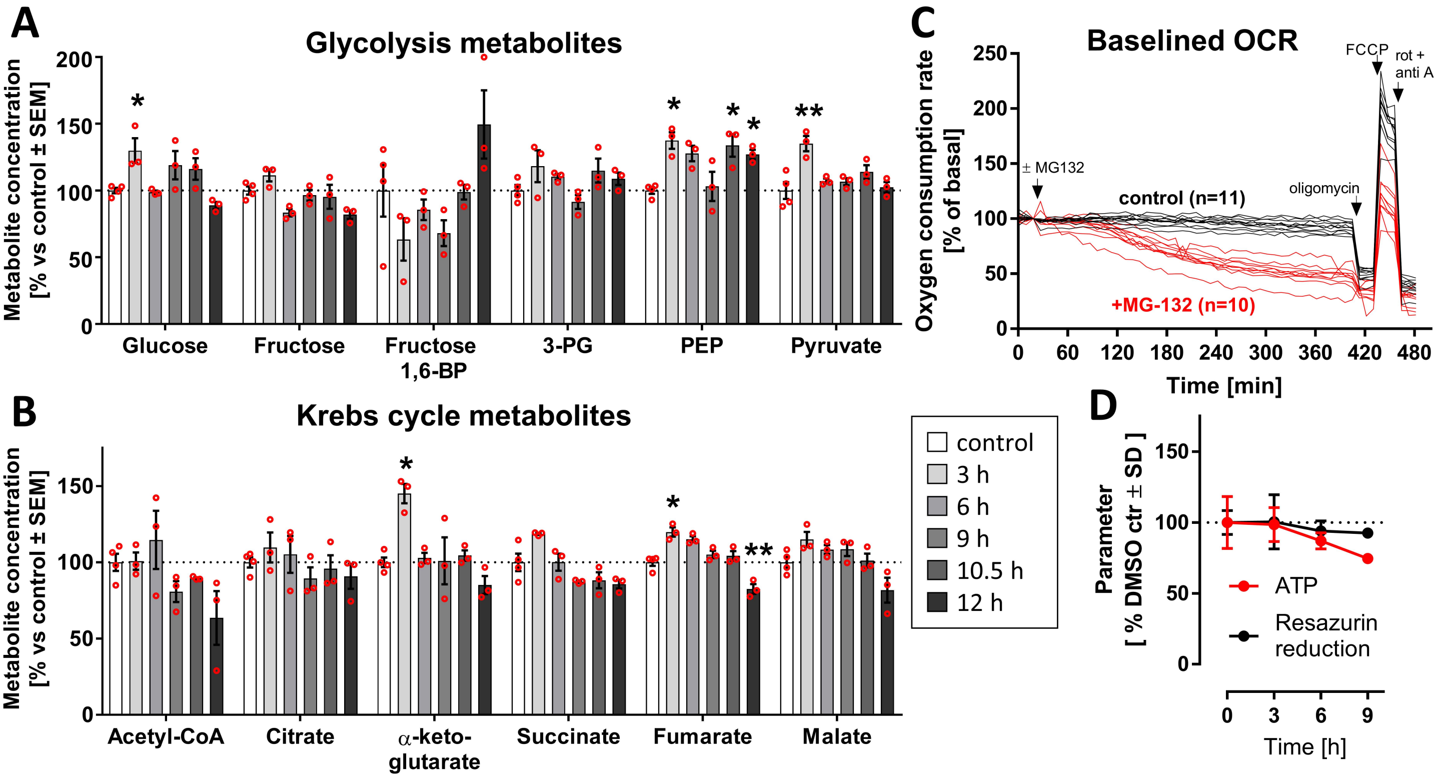

Fig. S12: Concentrations of all measured metabolites related to glycolysis and the citric acid cycle.

LUHMES cells were treated as described in Fig. 1 to obtain time-dependent metabolomics data, following addition of 100 nM MG-132. Data on all measured glycolysis and tricarboxilic acid metabolites were extracted from the metabolome data matrix (Suppl. Table 1.2). MG-132 exposure time-dependent changes of (**A**) glycolysis and (**B**) Krebs cycle metabolites. (**C**) The oxygen consumption rate (OCR) was measured for 6 h after 1 µM MG treatment, then the “Mito Stress Test” was performed. Each trace was obtained from one culture well. The data were pooled from two fully separate experiments. (**D**) Quantification of viability parameters (total ATP and resazurin reduction) over time. Metabolite data are means ± SEM of independent replicates. For MG-132 treatments, 3 different samples were analyzed. For controls (DMSO), 4 replicates were prepared to provide for more robust baseline data. * *p*-value ≤ 0.05; ** 0.001 < *p*-value ≤ 0.01; Acetyl-CoA, acetyl-coenzyme A; Fructose 1,6-BP, Fructose 1,6-bisphosphate; 3-PG, 3-phosphoglycerate; PEP, Phosphoenolpyruvate; rot, rotenone; anti A, antimycin A.

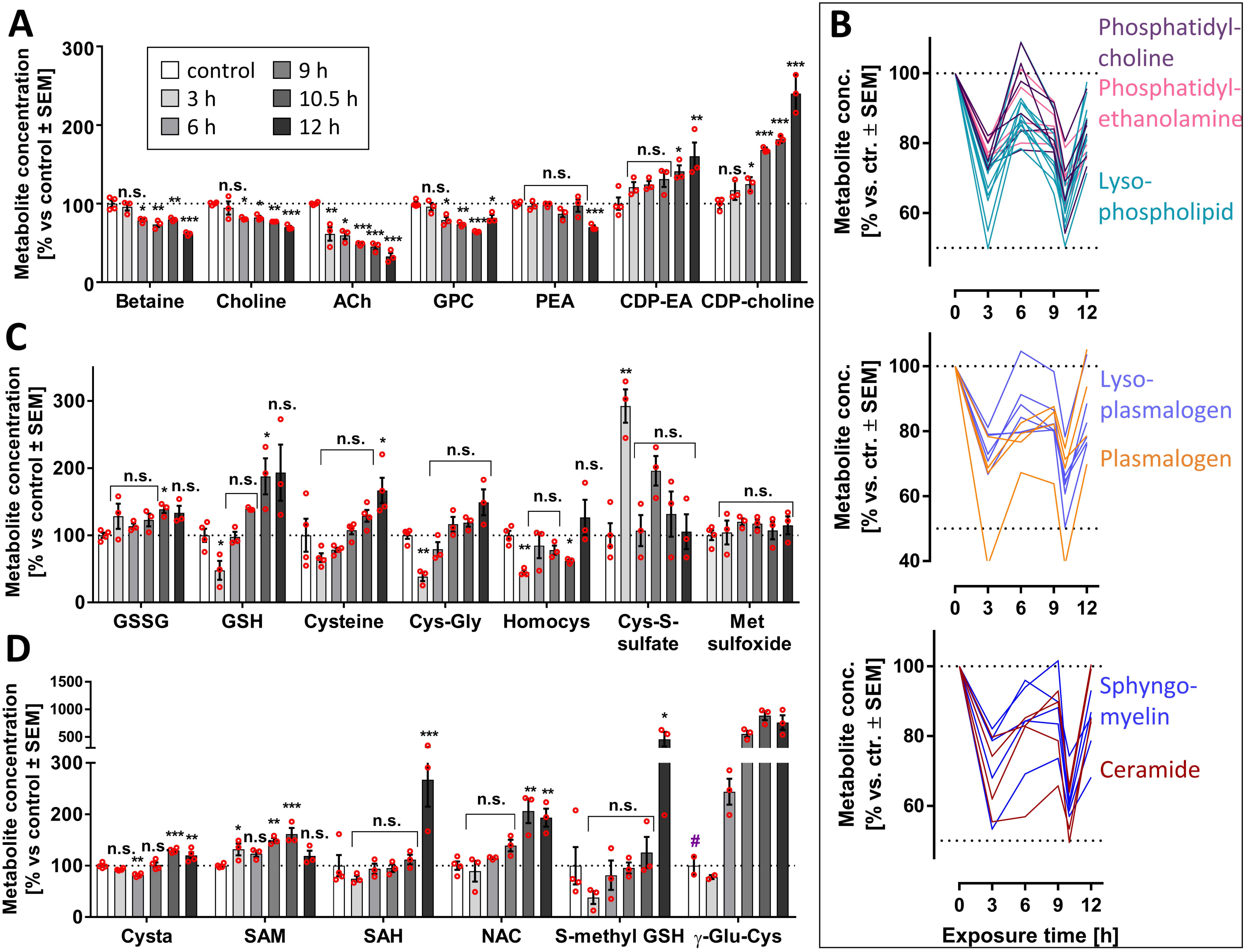

Fig. S13: Change of oxidative stress- and lipid-related metabolites over time.

LUHMES cells were treated as described in Fig. 1 to obtain time-dependent metabolomics data, following addition of 100 nM MG-132. Data on all measured oxidative stress- and lipid-related metabolites were extracted from the metabolome data matrix (Suppl. Table 1). Data for cysteine and cystathionine were obtained by amino acid analysis. MG-132 exposure time-dependent changes of lipid metabolites (**A**, **B**) and GSH synthesis intermediates (**C**, **D**). Metabolite data are means ± SEM of independent replicates. For MG-132 treatments, 3 different samples were analyzed. For controls (DMSO), 4 replicates were prepared to provide for more robust baseline data. * *p*-value ≤ 0.05; ** *p*-value ≤ 0.01; *** *p*-value ≤ 0.001; n.s., not significant; #, absence of statistical data (only 2 out of 4 replicates of the control were measured); ACh, acetylcholine; CDP-choline, cytidine diphosphate-choline; CDP-EA, cytidine diphosphate-ethanolamine; Cys, cysteine; Cysta, cystathionine; GPC, glycerylphosphorylcholine; GSH, oxidized glutathione; GSSG, reduced glutathione; Homocys, homocysteine; Met sulfoxide, methionine sulfoxide; NAC, N-acetyl cysteine; N-carb-L-Asp, N-carbamoyl-L-aspartate; SAH, S-adenosylhomocysteine; SAM, S-adenosylmethionine.

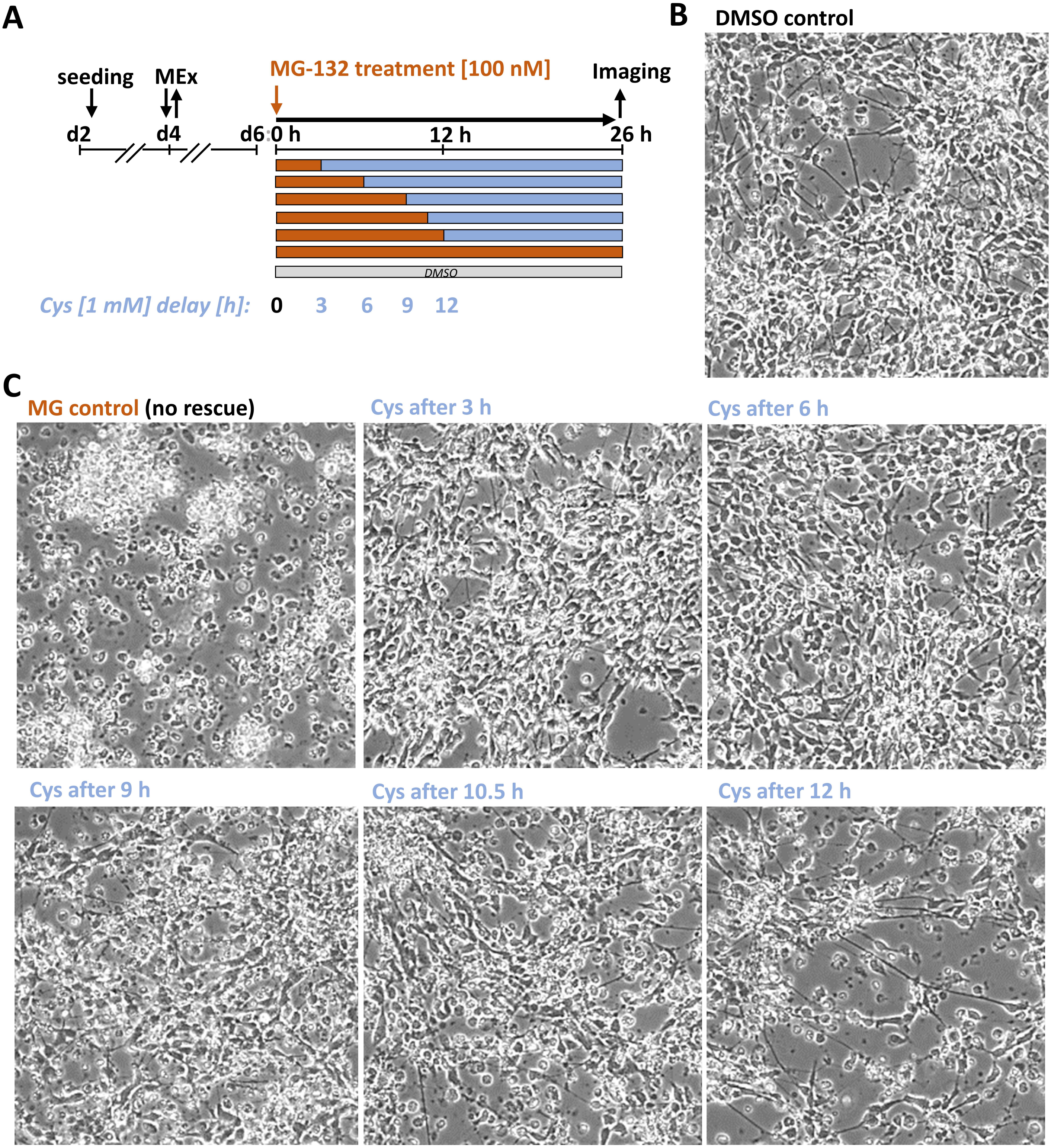

Fig. S14: Rescue time window of proteasome-inhibited LUHMES cells by using a thiol supply

(**A-C**) In parallel to the samples prepared for metabolomics, additional LUHMES dishes were rescued by addition of 1 mM cysteine (Cys) after 3, 6, 9, 10.5 and 12 h of treatment with MG. These were imaged at 26 h, along with the MG (positive) and DMSO (negative) controls, to visually assess the cell viability. MG, MG-132.

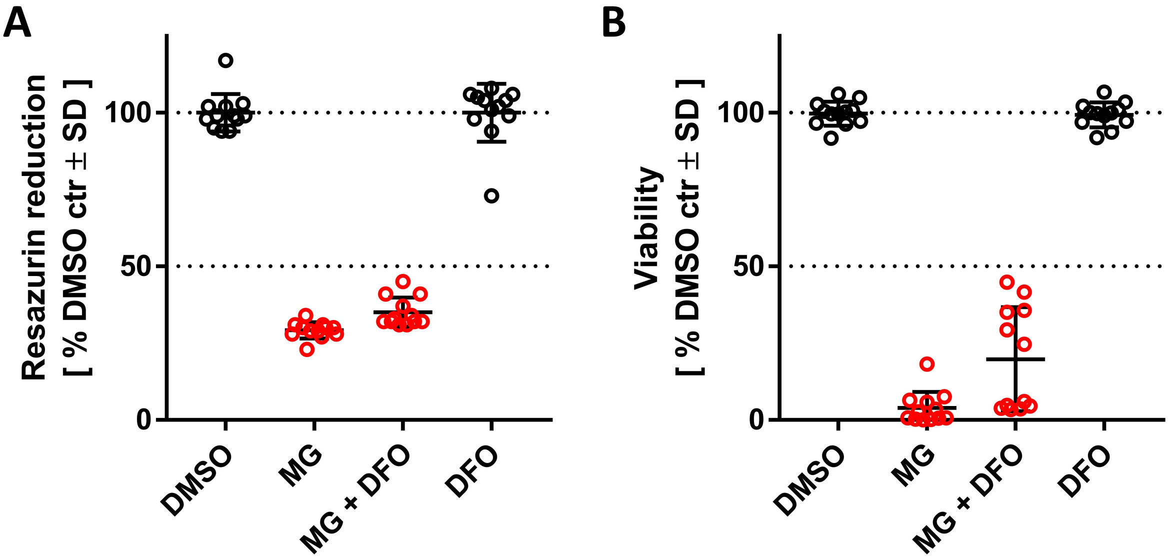

Fig. S15: Effect of iron chelation on MG-132-induced neuronal cell death

(**A-B**) On day 6, separate wells containing LUHMES cells were treated with either 100 nM MG, 50 µM deferoxamine (DFO) or a combination of both. Viability was assessed 24 h later by resazurin reduction to resorufin (**A**) or by Calcein/H-33342 co-staining (**B**) and then normalized to the DMSO control. Results are means ± SD of 12 biological replicates. MG, MG-132.
